# Individuals’ attitudes toward digital mental health apps and implications for adoption in Portugal: web-based survey

**DOI:** 10.1186/s12911-024-02488-1

**Published:** 2024-04-18

**Authors:** Diogo Nogueira-Leite, Manuel Marques-Cruz, Ricardo Cruz-Correia

**Affiliations:** 1 Health Data Science Ph.D. Program, Faculty of Medicine of the University of Porto, Porto, Portugal; 2 Department of Community Medicine, Health Information and Decision-Making, Faculty of Medicine of the University of Porto, Rua Dr. Plácido da Costa, Porto, 4200-450 Portugal; 3 Center for Health Technology and Services Research, Porto, Portugal; 4 Nova School of Business and Economics Health Economics & Management Knowledge Center, New University of Lisbon, Lisbon, Portugal

**Keywords:** Mobile health, mHealth, Digital health, Apps, Technology acceptance, Adoption, Mental health, Mental health services, Community mental health services

## Abstract

**Background:**

The literature is consensual regarding the academic community exhibiting higher levels of mental disorder prevalence than the general population. The potential of digital mental health apps for improving access to resources to cope with these issues is ample. However, studies have yet to be performed in Portugal on individuals’ attitudes and perceptions toward digital mental health applications or their preferences and decision drivers on obtaining mental health care, self-assessment, or treatment.

**Objective:**

This study aims to understand the determinants of digital mental health applications use in the Portuguese academic community of Porto, along with potential adoption barriers and enablers.

**Methods:**

A cross-sectional, web-based survey was delivered via dynamic email to the University of Porto’s academic community. Data collection occurred between September 20 and October 20, 2022. We used structural equation modeling to build three models, replicating a peer-reviewed and published study and producing a newly full mediation model shaped by the collected data. We tested the relationships between use of digital mental health apps and perceived stress, perceived need to seek help for mental health, perceived stigma, past use of mental health services, privacy concerns, and social influence.

**Results:**

Of the 539 participants, 169 (31.4%) reported having used digital mental health apps. Perceived stress and a latent variable, comprising perceptions of mental health problems and coping strategies, were positively associated with mental health app use, while privacy concerns regarding one’s information being accessible to others were negatively associated. Perceived stigma, need to seek help, and close relationships did not have a statistically significant direct effect.

**Conclusions:**

These findings can inform product and policy development of new, better-targeted digital mental health app interventions, with implications for researchers and academia, industry, and policymakers. Our study concludes that, to maximize adherence to these apps, they should have low to no financial charges, demonstrate evidence of their helpfulness and focus on the timely delivery of care. We also conclude that to foster digital mental health app use, there is a need to improve mental health literacy, namely regarding self-awareness of one’s conditions, acceptable stress levels, and overall behavior towards mental health.

**Trial registration:**

RR2-10.2196/41040.

**Supplementary Information:**

The online version contains supplementary material available at 10.1186/s12911-024-02488-1.

## Introduction

### Background

The lack of mental health, or a high prevalence of mental disorders, is associated with lower levels of quality of life [1, 2], life satisfaction [3, 4], and physical health [5], thus constituting a limiting factor to most aspects of human activity and autonomy [6].

Portugal presents worrisome mental health indicators in terms of disease cases and disability-adjusted life years (DALY): mental health disorders in 2019 were estimated at 19.27% of disease cases and 8.27% of DALY. The statistics for anxiety and depressive disorders were, respectively, expected to be 2.58% and 3.16% of total DALY and 9.08% and 5.88% of disease prevalence [7]. A summary of the comparison with the global and European Union (EU) landscape is available in Table 1.

**Table 1:** Share of DALY and disease prevalence (in percentage points) per condition and geography

	World		EU		Portugal	
	DALY	Prevalence	DALY	Prevalence	DALY	Prevalence
Mental disorders	4.92%	13.04%	6.65%	15.34%	8.27%	19.27%
Depression	1.84%	3.76%	2.42%	4.6%	3.16%	5.88%
Anxiety	1.13%	4.05%	1.69%	5.82%	2.58%	9.08%

Data source: Institute for Health Metrics and Evaluation (IHME). GBD Compare Data Visualization. Seattle, WA: IHME, University of Washington, 2020

The data for Portugal shows the magnitude of the existing problem, and the difficulties in accessing psychological and psychiatric care are evident. Waiting times for a psychiatry consultation ranged from 10 to 349 days in the 91 institutions of the Portuguese National Health Service that reported them from September to November 2022 [8]. The timeframe for a psychology consultation in the 12 institutions that reported it for the same period ranged from 15 to 148 days.

Considering that most depression and anxiety cases, albeit responsible for most of the disease prevalence of mental disorders, are classified as non-priority cases, waiting times can be expected to range from 30 to 349 days. It is likewise important to emphasize (i) the geographical asymmetries, (ii) the concentration of waiting times on higher bounds (e.g., above 100 days of waiting time), and (iii) the criticality of timely delivery of mental healthcare.

The limited access is further strangled by a share of out-of-pocket payments at about double the EU average [9, 10], making access to private sector providers a privilege for the few who can pay it.

The momentum of mental health is visible in two other contexts: digital health applications (apps), where there is ample penetration of apps for mental health purposes, and academic communities, where mental diseases are more prevalent and several experts have alerted to the situation at hand [11–13]. Various studies have been conducted to understand digital interventions’ impact in academic settings [14–19].

### Digital health applications

Digital health apps promise to make healthcare more accessible and personalized by delivering healthcare in constrained contexts, namely remote locations, lack of workforce resources, or insufficient transport infrastructure [20–23]. According to IQVIA’s Digital Health Trends 2021 [24], over 350,000 health apps are available in various app stores, 110 of which with over 10 million downloads.

The same report highlights that mental health, cardiovascular, and diabetes condition management apps account for nearly 50% of disease-focused applications in app stores. The number of downloads and variety of apps allowed many to conclude that there is apparent demand at the consumer level.

The scarcity of clinical and technical validation, and to a great extent of robust scientific evidence, is among the most significant perils digital health apps present [25]. Even though mental health is one of the disease areas where apps have proliferated, they can be misleading, as many of the tools currently being used for mental health fit into mindfulness or wellness categories, not being necessarily apps specifically designed to support patients in detecting or controlling specific diseases (e.g., anxiety or depression).

The diversity and volume of digital health apps in the mental health field add to the argument that this is one of the disease areas urgently needing widespread access due to its burden of disease and impact on health and health-related quality of life. And if no new intervention in health gains traction without individuals adhering to it, the volume and variety of mental health apps and related downloads is an argument that there is a market for it, and individuals are at least willing to test this approach.

### Mental health determinants and the academic community

Mental health problems affect young individuals disproportionately. OECD’s Health at a Glance 2022 for Europe [26] estimated that the COVID-19 pandemic significantly impacted young people’s mental health (defined as those whose ages ranged between 15 and 29 years old), with the rate of depressive symptoms more than doubling in several countries. Furthermore, around half of them faced unmet needs for mental health care both in 2021 and 2022, a rate about double that of all adults.

The report further emphasizes that young people with pre-existing and severe mental health issues reported worsening of their symptoms during the pandemic, that mental distress remains high into 2022, and that the pandemic highlighted linkages between income, inequality, and mental health. All these problems must be taken into a context where most mental healthcare facilities were already stretched, and severe disruptions in mental healthcare delivery were registered.

The academic community has been infamous regarding the mental health status of students, teachers, and administrative staff [27–30]. Taking into consideration Portugal’s worrisome mental health indicators, the pandemic’s detrimental effect on these indicators, and the alerts about the mental health problems in the country’s academic communities [29–33], one could argue that the academic community of one of the country’s largest universities is a good place to understand whether digital mental health apps (DMHA) could be a relevant resource to increase access to mental health resources for the wider population.

We anticipate this to hold as this community is expected to (i) reflect the urgent need for mental health resources and (ii) mimic the behavior of the section of the population most likely to adopt it (due to familiarity with smartphone usage, age, and literacy, among other factors). Despite recognizing that these characteristics are not fully replicable to the Portuguese population due to age structure, gender, and literacy levels, we expect our study’s results to help tailor specific interventions from academia, industry, and government in the academic context, as well as to pave the way for further research at the population level.

We anticipate that specific determinants of mental health in college students will be important for the analysis done in our study for DMHA use. Namely, we hypothesize that students having good social relationships with close ones and feeling comfortable about sharing their concerns with them [34–36], namely family and partners, is an essential affective association that may condition DMHA utilization. We also consider that the individual’s perception of their need to seek care due to having a diagnosed mental health problem or having a specific problem [35–38] is an important aspect to identify, and that may help explain their attitude and behavior toward DMHA in a more technologically enabled population [39].

Moreover, we hypothesize that another important factor in ascertaining these expectations and perceptions might be whether, in an era of ever-increasing connectivity and access to information, some already have certain coping or mental health-enhancing strategies in place [40–42]. Art therapy, comprising painting, drawing, coloring, and photography, can be effectively used in coping leisure strategies to aid in managing mental health.

In addressing the determinants of Digital Mental Health Application (DMHA) use within the academic community of Porto, our study proposes the following refined hypotheses:

1. H1: The strength and quality of social relationships, particularly the comfort level in discussing mental health concerns with close ones, such as family and partners, are positively associated with the utilization of DMHA. This hypothesis emphasizes the role of social support in the acceptance and use of digital mental health solutions.
2. H2: The individual’s perceived need for mental health care, influenced by either existing mental health diagnoses or specific psychological challenges, is a critical determinant of DMHA adoption. This highlights the importance of personal health awareness in technology acceptance.
3. H3: Engagement in coping strategies, including traditional and creative methods like art therapy, is indicative of a higher likelihood to adopt DMHA. This suggests that individuals already taking active steps to manage their mental health are more inclined to explore and use digital health interventions.

While several articles have been published regarding the use of DMHA, most such efforts have been trials of the effectiveness of already developed DMHA [16, 19, 43–46]. Borghouts et al. [47] have applied a questionnaire to college students regarding the potential use of DMHA and associated perceived barriers. In that sense, applying the same questionnaire, with due adaptations, to a Portuguese academic community would build on the relevant perceptions already identified and insights on the mental health status of this population while allowing for a direct comparison with a different college population.

To the authors’ knowledge, no studies have been performed in Portugal on individuals’ perceptions of DMHA, their preferences and decision drivers on obtaining mental health care, mental health self-assessment, or their perceptions of mental health and its treatment. Our study aims at bridging these gaps by mapping the expectations of the demand side of digital mental health apps. Our work may further provide necessary information for researchers, academia, industry, and policymakers on how to leverage DMHA as a tool to increase access to mental health care while ultimately contributing to reducing the burden of disease associated with mental health disorders.

## Objective

This paper aims to understand individuals’ attitudes toward DMHA in the Portuguese context. Participants were questioned regarding perceived benefits, barriers to adoption, and potential ways of supporting the adoption of DHMA.

## Methods

### Study design

The study is based on the structure outlined in a previously published research protocol [48]. It replicates the paper by Borghouts et al. [47] with adaptations to determine the factors that impact the acceptance and use of DMHA in Portugal, using the academic community of Porto as a proxy. It is an attempt to provide a clearer, if limited, landscape of what policymakers and product developers should expect regarding the demand characteristics for these tools.

Given that the sample is obtained from one academic community of a specific university in the north of Portugal, among other issues that arise from convenience sampling (such as potential biases regarding gender representativeness), the generalizability of results to the overall Portuguese population is expected to be limited. Nevertheless, we expect our findings to be, at least, generalizable to university populations in Portugal. Furthermore, we expect this to be the best possible proxy to the more digitally literate segments of the general population (and, thus, those more likely to try DMHA), given the overall levels of digital literacy, education, and age of the academic community’s population [39]. We first applied a questionnaire resulting from an adaptation of Borghouts’ implemented questionnaire to the Portuguese context. The questionnaire was delivered through the University of Porto’s dynamic e-mail function to maximize our reach potential to the entire academic community of the University. We then described the sociodemographic characteristics of the sample. We established a comparison with the replicated study by highlighting the differences in the methodology used and the results obtained. Our team then outlined the structural models we developed and their specificities.

### Questionnaire

We took the final survey questionnaire available on Borghouts [47] (Additional file 1) and translated it to Portuguese through a licensed translator. This translation was delivered to selected members of the academic community – five students and five teachers – for review. These members were asked to focus their feedback on how to calibrate the questionnaire to (1) reflect important questions to ask regarding the use of digital health tools by individuals and (2) a Portuguese mental health care context, particularly that for the screening of anxiety and depression.

Obtained feedback was incorporated to produce a final online survey questionnaire for this study (available in English in Additional file 2), except for the used pre-validated scales, where no major changes were made.

The final version was divided into 6 parts, namely demographic information, smartphone use, mental health apps use and perceptions, use of healthcare resources, stress, well-being and mental health experiences and mental health perceptions. Prevalidated scales used on Borghouts et al. were included in our study in their translated versions and were measured as follows:

- Perceived stress was measured with the 7-item version of the College Student Stress Scale (scored 1–5 in each item);
- Perceived need to seek help because of problems with mental health in the past 12 months was measured by a dichotomous response item;
- 2 dichotomous (yes or no) response items were used to identify past use of professional mental health services, such as a counselor, for mental health concerns in the past 12 months, where an answer “yes” to any of the two questions meant past use of health resources;
- Perceived stigma was measured using 9 statements from the Perceived Stigma subscale of the Depression Stigma Scale (scored 0–4 in each item);
- Social influence was measured using three statements (e.g., “People who are important to me think I should use mental health apps”);
- Privacy concerns were measured using 6 statements (scored 1–5 each) originally adapted by Borghouts et al. to refer to mental health apps specifically. These statements were further divided into two privacy constructs, with three questions each.

The survey questionnaire was pretested by five different colleagues to determine completion time and identify shortcomings. Completion time was estimated to be between twelve and twenty minutes. No shortcomings were identified.

The survey’s link was circulated via dynamic email by the University of Porto’s Communication Department. This tool allowed us to reach the complete total of the academic community – students, teachers, and administrative staff. The first communication email was sent out on September 20, and a reminder followed on October 6. The period for answer collection ran from September 20 to October 20, 2022. A total number of 33,668 email addresses received both emails. The platform used was Inqueritos@UP, the University of Porto’s internal survey manager by LimeSurvey, and the survey adheres to and is reported following the Checklist for Reporting Results of Internet E-Survey (CHERRIES) guidelines.

**Fig. 1:**
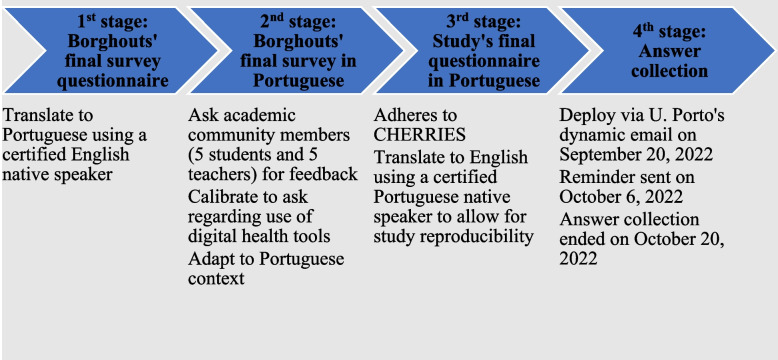
Survey adaptation and communication workflow

To maximize response, the only inclusion criterion was to have an active registration with the University of Porto domain, defined as the ability to receive the survey invitation email. No exclusion criteria were introduced, and no financial incentives were offered. Figure 1 summarizes the survey’s adaptation and communication workflow.

Gathered data were analyzed according to the methods used by Borghouts et al. to allow for maximum comparability between results. We mapped the summary of modifications between the initial questionnaire (i.e., Borghouts’) and the final survey questionnaire used, and made it available in Additional file 3.

### Participants

A total of 968 participants started the survey, and 539 completed it, resulting in a response rate of 1.7% and a 55.7% completion rate. The survey participants had a mean age of 24.3 years (SD 11.1); 68.3% identified as female and 26.9% as male. Of the 539 respondents, 506 (93.9%) identified as White, 12 (2.2%) identified as having more than one ethnicity, and 3 (0.6%) identified as Asian.

Most reported being unemployed (72.5%), living with their families (59.9%), and being single (63.3%). Some faculty members (7.1% of the sample) and administrative staff (2.8% of the sample) answered the survey. This sample thus meets the goal of encompassing the academic community beyond the traditional scope of students only. Of the 539 participants, 77 (14.3%) had an annual household income of less than EUR €10,000. Most participants (77.7%) had health insurance, but only 27.1% of those with insurance were sure that their plan provided coverage for mental health services. Further demographic characteristics are shown in Additional file 4.

Differences between participants who had used or were currently using DMHA (users) and participants who never used a DMHA (never-users) were also assessed (Additional file 5). DMHA users were significantly older (mean 25.6 vs. 20.9 years old, *p* < .001), with a higher proportion of females (74.0% vs. 65,7%) and other genders (10.7% vs. 2.2%) (*p* < .001). Significant differences were also found in enrollment status (*p* = .003), with full-time enrollment of 91.1% vs. 79.7% and faculty members of 1.2% vs. 8.1%; marital status (*p* = .003), with married participants of 4.2% vs. 16.0%; children whom they were responsible for (96.4% vs. 82.4%, *p* < .001); household income (*p* = .023), with 32.4% vs. 16.5% reporting an annual household income of less than EUR €10,000 and 40.0% vs. 49.2% reporting between €10.000 and €27.500; and presence of a disability (11.4% vs. 5.7%, *p* = .022). Employment status, race, living arrangements, homelessness, and health coverage were not significantly different between users and never-users.

Our study sample had a significantly higher number of females, full-time students, white people, and unemployed than Borghouts et al.’s sample. It also had a significantly lower number of homeless people and health insurance compared with Borghouts et al. A statistically significant difference was also found in maternal status and living situation. The age and existence of children and dependents or disabilities were not significantly different.

### Comparison with the replicated study

The survey followed the same structure as Borghouts et al. concerning Barriers to Mental Health Resources, Mental Health Problems, Mental Health App Use, Past Use of Professional Mental Health Services, Perceived Stress, Perceived Need to Seek Help, Mental Health Concerns, Perceived Stigma, Social Influence, and Privacy Concerns. More detail can be found in the primary survey instrument in Borghouts et al. Our final questionnaire in English is available in this study’s Additional file 2, and a summary of modifications is available in Additional file 3.

To reflect the hypotheses, we laid out in the introduction, we focused our study on the following items:

- Deriving from a multiple-answer question (Q31), we created a new variable where the existence of a support network was defined as usually talking to a family member or partner when feeling sad, anxious, preoccupied, or stressed.
- From question Q23a, we grouped participants into a new single variable defining participants reporting depression, eating disorders, obsessions, and compulsions interfering with daily activities or bipolar disorder as determinants of the perceived need to seek help given their prevalence in the academic community. These specific mental health problems were chosen because they had a weak correlation (defined as a correlation coefficient between 0.10 and 0.30) with each other.
- From question Q32, one other variable (“Coping Strategies”) was created that included individuals that reported spending time with pets (a strategy added to our questionnaire), social media, painting, drawing, coloring, and photography, or writing as leisure strategies used to aid in managing mental health.

### Structural models

Three models were developed: two replicating Borghouts et al. and a third model representative of the data obtained from our questionnaire.

For the replicated models, we used the same variable definitions, namely two privacy constructs and social influence as latent variables, since the items included were adapted from validated scales but have not been tested in prior work as a single scale beyond the work of Borghouts et al. Gender, age, perceived stress, past use of professional mental health services, perceived need to seek help, and perceived stigma were included as observed variables.

Our model was based on the direct effects model of Borghouts et al., including the variables with statistically significant relations with the use of mental health apps. On top of said model, we included coping strategies as an observed variable, and a latent variable (Perceived Mental Health Problem) derived from three observed variables, namely the grouped variable from Q23a, a self-reported mental health problem (Q41), and perceived need to seek help. We also expected to find a high correlation between coping strategies and perceived mental health problem and combined them into a single latent variable. The correlation matrices for these two latent variables are presented in Tables 2 and 3.

**Table 2:** Correlation matrix for perceived mental health problem

	Q23a	Q41	Perceived need
Q23a	1.000		
Q41	0.558	1.000	
Perceived need	0.415	0.475	1.000

**Table 3:** Correlation matrix for latent variable

	Coping strategies	Perceived problem
Coping strategies	1.000	
Perceived problem	0.523	1.000

The correlation matrix of the variables is displayed in Table 4. The correlation values further show that there were no strong correlations between the variables, except for perceived need and latent variable (unsurprising when considering that perceived need also contributed to the latent variable).

**Table 4:** Correlation matrix of model variables

	Perceived need	Perceived stigma	Close relationship	Perceived stress	Privacy construct 1	Latent variable
Perceived need	1.000					
Perceived stigma	0.226	1.000				
Close relationships	-0.079	0.028	1.000			
Perceived stress	0.000	0.000	0.000	1.000		
Privacy construct 1	-0.012	-0.009	0.003	0.000	1.000	
Latent variable	0.546	0.414	-0.144	0.000	-0.023	1.000

### Analysis

The model’s dependent variable was the participants’ mental health app use as a dichotomous (yes or no) variable. For the replicated models, the independent variables were perceived stress, perceived need to seek help (shortened in the Results section as perceived need), past use of professional mental health services (abbreviated in the Results section as past use of services), perceived stigma, social influence, and privacy concerns represented by two different constructs (numbered 1 and 2, respectively). Age and gender were added as covariables. Table 5 shows the names and descriptions of all variables included in our models.

**Table 5:** Name and description of model variables

Name	Borghouts	Description
User	User	Participants’ mental health app use
Perceived Stress	Perceived Stress	7-item version of the College Student Stress Scale originally included in Borghouts et al. and translated in our questionnaire.
Perceived Need	Perceived Need	A dichotomous response item originally included in Borghouts et al. and translated in our questionnaire.
Past Use of Services	Past Use of Services	2 dichotomous response items originally included in Borghouts et al. and translated in our questionnaire.
Perceived Stigma	Perceived Stigma	9 statements from the Perceived Stigma subscale of the Depression Stigma Scale originally included in Borghouts et al. and translated in our questionnaire.
Social Influence	Social Influence	3 statements originally included in Borghouts et al. and translated in our questionnaire.
Privacy Construct (1 and 2)	Privacy Construct (1 and 2)	6 statements (3 for each construct) originally included in Borghouts et al. and translated in our questionnaire.
Age	Age	
Gender	Gender	In our study, we included “other” gender.
Coping Strategies	-	Individuals that reported spending time with pets, social media, painting, drawing, coloring, photography, or writing to aid in managing mental health.
Perceived Problem	-	Perceived Mental Health Problem: latent variable defined by the self-reporting of depression, eating disorders, obsessions, and compulsions interfering with daily activities or bipolar disorder, a mental health problem and Perceived Need.
Latent Variable	-	Latent variable resulting from Perceived Problem and Coping Strategies.
Close relationships	-	Multiple choice question, regarding each person’s support network. Considered to have a support network of close relationships if either family or partners were included as people to whom they talk when feeling sad, anxious, worried or stressed.

In our extended model, a latent variable, including coping strategies and perceived mental health problem, was added to the model as an independent variable.

Structural equation modeling (SEM) tested the relationships between measured variables. The measurement models’ fit was evaluated with the comparative fit index (CFI), the models’ chi-square, the RMSEA, and the SRMR [49]. Maximum likelihood was used as an estimator for the structural equation models.

We compared the fit of our extended model with the full mediation and direct effect models based on the replicated study. Our extended model did not consider mediating effects of any variable. However, perceived need was included as a direct variable and inside perceived problem.

In our extended model, a latent variable was added as an independent variable, including non-clinical strategies and perception of mental health problems.

Full maximum likelihood was used to impute missing data on scales. Participants with missing data on dichotomous model variables (i.e., perceived need and past use of services) were excluded from the model. The software environment R (R Foundation for Statistical Computing) was used for statistical analysis, and the R package lavaan was used for the structural equation models and bootstrapping [50].

### Ethics approval

The Ethics Committee of the Faculty of Medicine of the University of Porto pronounced itself favorable to the research project on June 30, 2022 (Opinion 52/CEFMUP/2022).

Ethical considerations and safeguards for the study and its supporting documents (including the online survey) comprise the following, having received approval from the Data Protection Officer of the University of Porto:

- To preserve participants’ privacy, they will not be asked to provide any personally identifiable information. In addition, participants will not be tracked for having started or completed the survey, increasing privacy but limiting the possibility of reminders.
- Informed consent and consenting capacity: all potential participants (physicians and academic community members) will be given online written information on the study and its objectives and will be asked to provide consent (click-to-agree) that they are happy to participate, and that nonparticipation will not compromise their current roles. Participation in the study will be voluntary, and no inducements or incentives will be offered.
- Confidentiality: Any data/personal details that could potentially reveal the identity of individuals will be removed. Only anonymized, deidentified information will leave the place of origin. A database with responses will be maintained on a password-protected database. All research data will be stored on a password-protected desktop computer at the host organization. Study participants will be invited, through a link provided on the last page of the survey, to provide their name and electronic address to allow the research team to facilitate their receiving a synopsis of the study findings on publication. This list will be kept separately on a password-protected database and a password-protected desktop computer at the host organization. All data will be stored securely at the host institution and destroyed three years after the Ph.D. defense date in November 2023.
- General Data Protection Regulation: GDPR compliance will be adhered to in terms of the following:
  - ◦ Data privacy rights – participants will have the right to request information about their data throughout the research process.
  - ◦ Transfer of data – participants will be informed about the circumstances under which their data may be transferred and safety measures that will be taken to protect the data (e.g., data are encoded).
  - ◦ Retention of data – participants will be informed how long their data will be stored.

Inquéritos@UP stores survey data at U. Porto’s servers and thus it is not shared with external entities, constituting another layer of privacy protection. Further, the survey’s first page briefly explained the required data and the rationale for asking for it.

## Results

### Technological and mental health resources and concerns

Of the 539 participants in our study, 265 (49.2%) reported that they had experienced a mental or psychological illness, significantly higher than Borghouts’ 189 (37.8%) respondents. Regarding specific mental health issues, 347 (64.4%) of respondents reported anxiety concerns, and 309 (57.3%) participants reported stress-related problems. These issues, also among Borghouts’ top two mental health concerns, differed significantly in their study, with 207 (41.4%) and 219 (43.8%) participants reporting anxiety and stress, respectively.

The stigma score in our sample is higher and less dispersed than that of Borghouts’ sample: 23.9 (SD 5.4) compared with 22.4, respectively, suggesting higher perceived stigma. Moreover, 169 (31.4%) respondents to our survey currently use or have used a mental health app, compared with Borghouts’ 106 (21.2%). A full overview of the responses to the questionnaire can be found in Additional file 6.

### Barriers to mental health resources (non-app)

Some mental health resources, such as counseling and mindfulness or stress management workshops, are provided by the University of Porto and its Faculty of Medicine. During the process of survey adaptation described in the “Questionnaire” sub-section of “Methods”, several answer options for the barriers to accessing mental health resources were changed, limiting the comparability between both studies. The frequency of each perceived barrier to mental health resources from our sample can be found in Additional file 6.

The main barrier to mental healthcare is financial, with 201 persons (37.3%) feeling that constraint. The extent to which these resources might be helpful, along with self-doubts concerning the seriousness of one’s needs, gathered 145 (26.9%) and 142 (26.3%) of provided answers. It should be noted that 24.9% of the sample considered the waiting time to access mental healthcare resources too long, resulting in a barrier to the mental health resources offered by the University of Porto. Furthermore, the fact that stress is accepted as usual at college (22.6%) and believing the problem will improve by itself (19.3%) are belief-related issues that could prove challenging to shape. Finally, it is interesting that a significant minority does not have the time to access these resources (16.1%) or that it does not access them due to privacy concerns (16%).

### Important aspects of mental health apps, and activities people would like to do with digital mental health apps

In our survey respondents’ opinion (Additional file 6), the most critical aspect of using mental health apps was the app being free of charge (83.3%). This was also the most important aspect of Borghouts’ survey, registering a similar magnitude (85.8%). The assurance that personal information will be kept private and that parts of the app can be accessed online came in second and third, respectively, with 437 (81.1%) and 288 (53.4%) responses. Borghouts’ study, on the other hand, registered the same second-ranked option but differed on the third most-voted option (people on the app having similar mental health experiences to theirs): 45.6% of Borghouts’ sample believed this to be important, in contrast with 29.7% in our sample.

Regarding the most common activities participants would like to do on apps (Additional file 6), our survey’s respondents stated that working through negative emotions and thoughts would be the primary use (70.1%), similar to Borghouts’ survey (65.8%). The ranking order was also the same for the second and third most reported items. Identifying or recognizing symptoms was the second most reported answer option in both surveys, with our sample registering 63.5% and Borghouts 58%. Tracking symptoms was the third most reported option, with 54.9% in our survey and 47.8% in Borghouts’.

### Models based on Borghouts et al

We proceeded to elaborate on the direct and fully mediated effects models based on Borghouts et al.’s work.

**Fig. 2:**
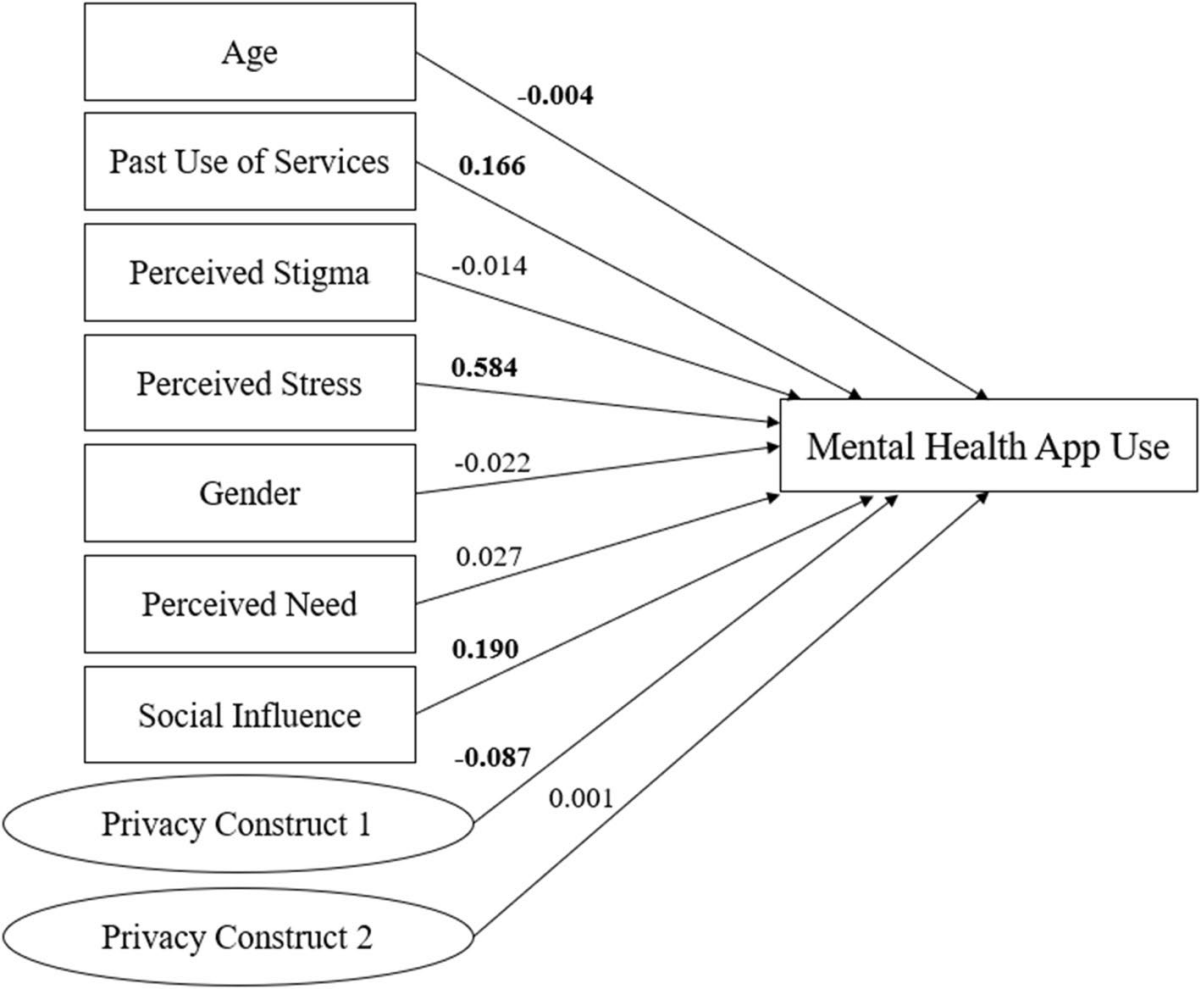
Direct effect model showing the path coefficients and levels of significance for relationships among variables. Coefficients in bold are statistically significant at *P*<.05; *n*=499

The standardized path coefficients of the direct effect model are shown in Fig. 2. The model fit indices were χ^2^_57_ = 173.118, SRMR = 0.067, and RMSEA = 0.064. The adjusted *R* ^2^ value of the model was 0.205.

**Fig. 3:**
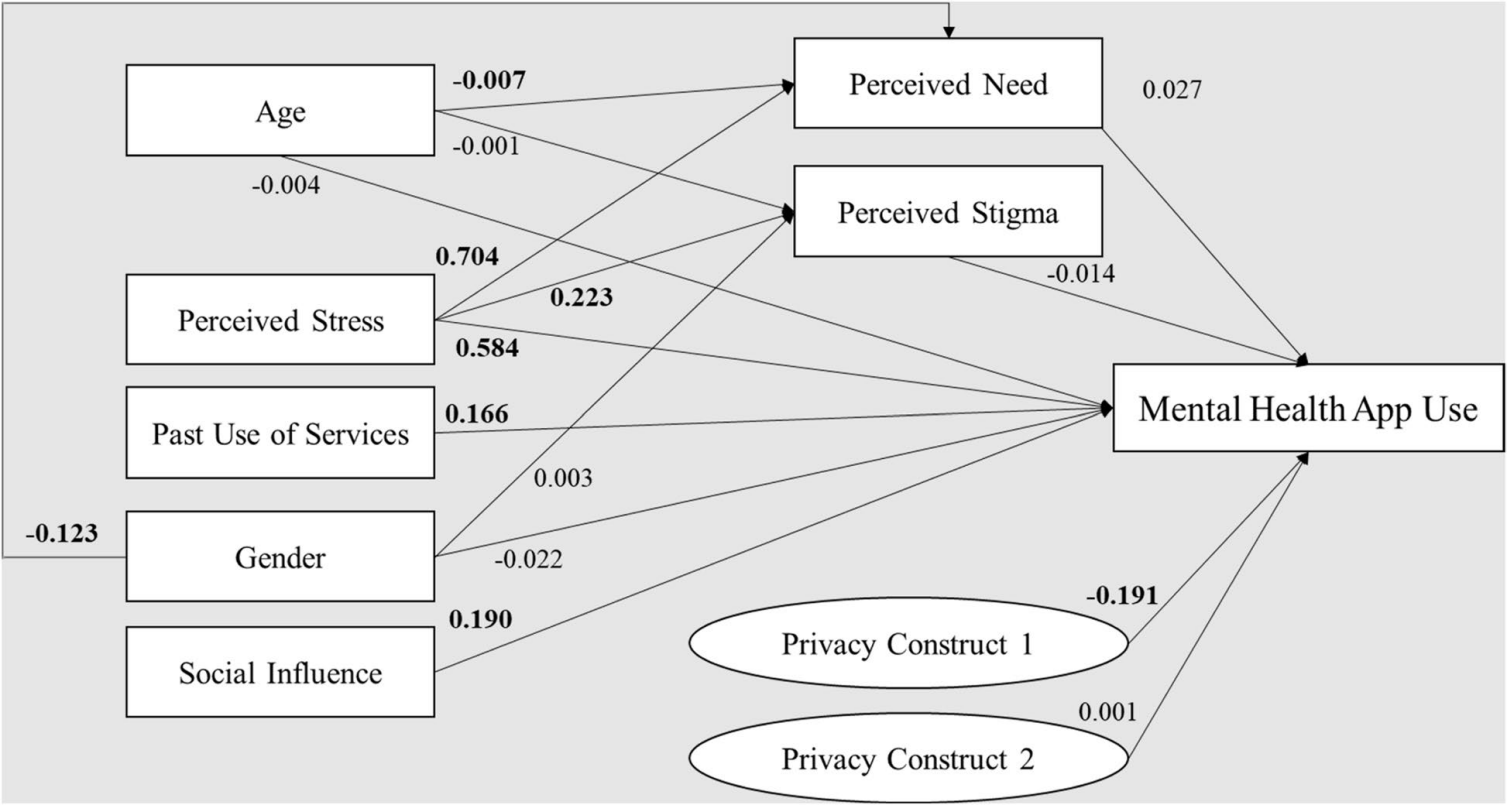
Full mediation model showing the path coefficients and levels of significance for relationships among variables. Coefficients in bold are statistically significant at *P*<.05; *n*=499

The standardized path coefficients of the full mediation model are shown in Fig. 3. The model fit indices showed an appropriate model: χ^2^_60_ = 264.901, SRMR = 0.045, and RMSEA = 0.051. The adjusted *R* ^2^ value of the model was 0.202.

Model fit and R-squared obtained for the direct effect model were comparable to those from Borghouts et al., but the same cannot be said of the full mediation model: our R-squared was considerably lower (0.202, vs. Borghouts’ 0.326), despite other fit indices being relatively similar.

### Model with additional MH determinants

Based on the previously detailed hypotheses regarding MH determinants, we developed a model to represent our data in this study. This research effort endeavored to build on Borghouts et al.’s work and elicit more information to more precisely depict the Portuguese context portrayed by this survey.

**Fig. 4:**
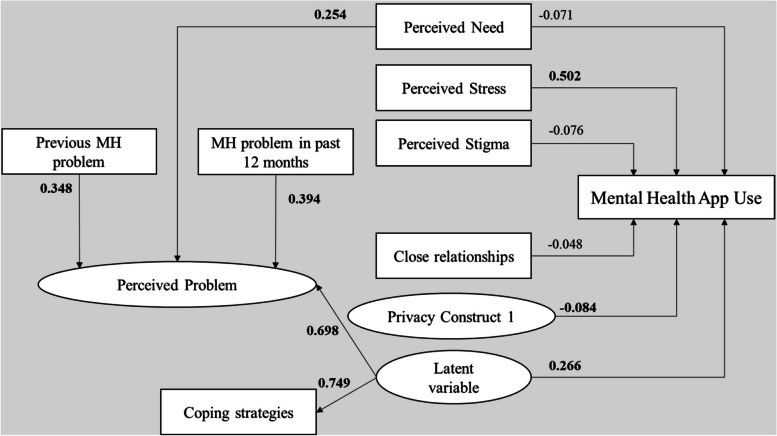
Extended mediation model showing path coefficients and levels of significance for relationships among variables. Coefficients in bold are statistically significant at *P* < .05; *n* = 499

The standardized path coefficients of the full mediation model are shown in Fig. 4. The model fit indices showed an acceptable model fit: χ^2^_69_ = 218.123, SRMR = 0.088, and RMSEA = 0.065. The adjusted *R* ^2^ value of the model was significantly higher than the results obtained via the mediated effects model of Borghouts et al.’s work (*R* ^2^: 0.419).

Table 6 compares the fit indices of our direct effect and full mediation models based on Borghouts et al. with those of our extended model, as shown in Fig. 4.

**Table 6:** Fit indices of the direct effects model and full mediation model

Statistic	Direct effects model (based on [47])	Full mediation model (based on [47])	Extended model
Chi-square (df)	173.118 (57)	264.901 (60)	218.123 (69)
SRMR^a^	0.067	0.045	0.088
RMSEA^b^	0.064	0.051	0.065
R2	0.205	0.202	0.419

^a^ *SRMR *Standardized root mean square residual

^b^ *RMSEA* Root mean square error of approximation

Our model showed that using mental health apps was significantly associated with perceived stress and Privacy Construct 1. DMHA use was also associated with a latent variable incorporating coping strategies and perceived mental health problems.

## Discussion

### Principal findings

This paper aimed to identify factors associated with mental health app use in the academic community of the University of Porto as a proxy for the Portuguese population and compare our results with Borghouts et al. Our results revealed that participants’ use of DMHA was associated with four factors: perceived stress and privacy concerns directly, and perception of mental health problem and having coping strategies in place through a latent variable comprising both.

The data’s descriptive analytics were like Borghouts’ except for the race breakdown (namely, the percentage the white population represented and, consequently, the lack of ethnic diversity found in Borghouts et al.). We also did not find statistical significance for perceived need for social influence, whereas Borghouts et al. did.

Our structural model also attained an *R* ^*2*^ of 0.419, which is almost 20 percentage points above the result achieved from the mediated effect model applied to our sample, and 10 percentage points above the same result obtained by Borghouts et al. in their sample. Based on our initial hypotheses, we were able to develop a model that can expand Borghouts’ work and provide a better explanation for the factors that contribute to DMHA use for our sample. It is noteworthy, however, that our full mediation model replicating Borghouts’ underperformed it in terms of R-squared.

### Perceived stress

Stress was the second most common mental health concern among participants (approximately 57% of participants experienced stress), just behind anxiety (64% of the sample vs. 41% in Borghouts). Our study’s results, like Borghouts’, indicate that the perceived stress level predicted mental health app use. Consequently, the more stress people in the academic community reported, the more likely they were to have used DMHA. We found this variable statistically significant and directly linked to DMHA use.

In contrast, Borghouts found it statistically significant only through a mediating effect of perceived need to seek help and not as a standalone variable. We posit, as Borghouts did, that this may represent a linkage between one’s use of DMHA and a potential benefit in stress reduction, which is supported by prior evidence [51, 52]. However, a causal link is still absent, and further research with that specific intent would be precious. This is reinforced by the widespread lack of robust scientific evidence of efficacy in most DMHA [16, 53].

### Latent variable

In our expanded model, the latent variable, which mediates the perception of a mental health problem and having coping strategies related to DMHA use, was demonstrated to be statistically significant and positively associated with DMHA use. This variable captures very diverse information. Its result means that a higher perception of having a mental health problem, a prior diagnosis of mental illness (either throughout one’s life or in the past 12 months), having used DMHA in the past, or having leisure coping strategies in place makes one more likely to use DMHA.

We hypothesized this variable based on a set of assumptions. Firstly, the available scientific evidence points to previous mental illness as a predictor of mental illness onset [54, 55]. This might lead one to seek help, as can an increased perception of having a mental health problem, e.g., trying the most downloaded DMHA or obtaining a diagnosis through a symptom checker. Secondly, past use of DMHA, especially if the experience was positive, may reinforce behavior or make someone more familiar with this intervention and thus more prone to do it again. Borghouts et al. found a positive and statistically significant relation between past use of services and DMHA use in their full mediation model, as we did while replicating it.

Finally, we assumed that having leisure coping strategies in place might reflect higher self-awareness concerning mental health and methods to enhance it. Thus, people with higher use of leisure coping strategies might be more inclined to use DMHA to maintain their mental health. Our results seem to point to our hypotheses being valid, but they did not have the same degree of association standalone. Still, the result of aggregating them in a latent variable warrants a further detailed study of these variables and their interconnections.

### Privacy

In our expanded model, the first privacy construct, i.e., related to one’s information being visible to others, demonstrated to be statistically significant and negatively associated with DMHA use, just as in Borghouts’ study. This result means that if someone is concerned about their information being visible to others that person would be less likely to use a DMHA. Curiously, the magnitude of the relationship is almost four times higher compared to Borghouts et al., albeit privacy concerns rank quite low in terms of stated barriers to DMHA use.

When specifically asked about privacy, respondents maximized its importance, while in confrontation with other barriers (e.g., financial burden), privacy assumed a relatively smaller weight. Although the literature and various anecdotal cases demonstrate how privacy policies and practices in DMHA are important [18] but often frail [16, 56], this result might illustrate the relative premium people place on keeping their personal information private, as well as how much of an obstacle it is to DMHA use. Moreover, we registered a non-significant relation for the second privacy construct (personal information being subsequently used), just as in Borghouts’ study.

These results allow us to fundament the need to have a deeper understanding of what privacy means to people and how they perceive it to be preserved, as beliefs with no technical background may result in a future loss of trust in DMHA or other digital interventions. It also begs the need for more studies on digital literacy and how it relates to privacy perceptions and attitudes.

### Other important findings

Self-reported mental illness registered significant differences between our sample and Borghouts’ sample: 49% of our sample said they had a mental illness compared with Borghouts’ 38%. Regarding their prevalence, we found that our sample had the most common mental illness, anxiety (64%), followed by stress (57%). In Borghouts’ sample, the rates for the same diseases were 41% and 44%, respectively. This very significant difference may be explained by having different populations under study. Given the pandemic’s impact on young people and digital health tool utilization [26], it cannot be excluded that this difference may be partially explained by the cross-sections performed during and after the pandemic’s most critical stage.

Another of the most significant differences we found between our sample and Borghouts et al.’s was the self-reported perceived need to seek help: 13% said they perceived that need against Borghouts 44%. Furthermore, concerning the use of professional services in the past 12 months, 43% of our sample reported having used such services, while Borghouts’ rate for the same variable was 23%. DMHA use was relatively similar except for the segment of past users of DMHA: 25% of our sample reported having used them in the past, contrasting with Borghouts’ 14%. All these issues could be fitted in the access to care category.

We hypothesize they may be due to differing capabilities in accessing mental healthcare and its consequences in lowering the perceived need to seek help – assuming the quicker one gets help, the lower the perceived need to seek help. Moreover, we hypothesize they may be due to two factors: the pandemic impact on access to digital healthcare and the sources of financial protection in illness.

Regarding the first factor, the difference in the past use of DMHA may be partially attributable to having tried DMHA as a means of coping with the pandemic, which is corroborated by the existing evidence on download numbers [24]. On the other hand, the significant difference in the percentage of people with health insurance between samples – 65% of our sample had it, compared with Borghouts’ 80% – is likely owed to different health system organizations. In Portugal, the National Health Service, funded by general taxation, is the central piece of the system, mainly providing free access at point-of-care and thus having low financial barriers to seeking healthcare. In this context, health insurance is often used as an ancillary means to speed up healthcare access by contracting with privately-owned healthcare providers. In the United States of America, given the centrality of health insurance in the system (either publicly or privately funded), there might be more significant financial barriers to seeking care, including mental health.

### Implications

Previous work has shown that despite interest in mental health apps among students, the use of these apps can be limited [57–59]. Building on the earlier work whose methodology we replicated, our findings allowed us to uncover various factors associated with DMHA use in the academic community and, more particularly, its students. We hope this work helps build more robust development and implementation efforts of digital mental health interventions, particularly app-based ones, on campuses or similar contexts.

First, it must be highlighted that no doubts remain concerning the importance of the academic community having the necessary mental health resources to tackle its very significant needs. The prevalence of mental illness and the identified mental illnesses are perfectly in line with the alerts regarding the COVID-19 pandemic’s impact on mental health [29]. There must be decisive action and involvement of all stakeholders in designing these interventions to best answer the community’s needs.

Secondly, we have discovered a strong association between privacy and DMHA use. This trait’s prevalence and relative weight make it critical to keep in mind when developing and implementing successful DMHA-based interventions. It is also important to foster digital literacy regarding privacy best practices to generate trust in digital tools, particularly those applied to mental healthcare.

Thirdly, the fact that the latent variable, while containing such diverse information, presents a considerable value leads us to believe that it is essential to look closely at the factors mediated through it. All of them are associated with prior mental illness and past use of services. This suggests that interventions should be tailored to efficiently triage between those who have already experienced mental health issues and those who have not, as well as constituting a pleasant and easy-to-use entry point to provide practical, evidence-based mental health-promoting resources.

Fourthly, one needs to carefully ponder the importance of social determinants of mental health [60] to properly engage the academic community, specifically its students, with DMHA use. Even though social influence was not statistically significant, mental illness is complex and involves issues such as poverty, low incomes, emotional strain, and poor housing conditions. Due to the pandemic and insufficient responses, many of these issues have increased in Portuguese society in recent years [33, 61]. Any digital intervention, app-based or not, must consider social determinants and adequately adjust its user interface and experience if it wishes to be successful.

Finally, all the above may have important implications for connected areas of knowledge and care delivery, namely public health, medical work, and social work and counselling: all these roles can be made more effective if they take the above implications into consideration. By tailoring these apps to better suit the perceptions and overcome identified barriers to their use may, for example, enable the deployment of public health interventions such as screening for common mental disorders in a scalable and adaptable manner. By the same token, the impact of properly designed and delivered DMHA on medical work may improve and enlarge access to diagnosis, treatment, and follow-up, as well as emergency, real-time consultations. At last, social work and counseling may be deployed in a more personalized manner and at a lower cost per episode, allowing for a better allocation of resources to high-priority cases without disregarding the long tail of low-priority, but high disease burden, cases.

### Limitations and future work

This study has several limitations. First, the sample came from one academic community of a specific university in the north of Portugal. This means that the generalizability of the results is limited. Nevertheless, we expect our findings to be generalizable to university populations.

Another of our sample’s significant limitations is gender representativeness: while 68.3% of our sample were female respondents and 26.9% were male respondents, with the remaining gender self-identifications amounting to 4.8%, 54% of the students in the University of Porto are female [62], with no further gender breakdown. This element also limits the generalizability of obtained results. Two other issues that should be borne in mind are the difficulty recruiting students and the wider academic community [63] and the ethical impact of DMHA. Some work has been done in this field [64], but much remains to be done.

A nationwide survey that addresses the above-mentioned challenges would be a welcome addition to the body of evidence surrounding the issue of DMHA-based interventions, enabling a more accurate mapping of attitudes and expectations from academic communities. However, it should also be recognized that full institutional support is necessary to succeed.

Additionally, we focused on presenting and discussing a specific model that expanded a prior one to test the effects of six factors on mental health app use. Despite allowing for a significant degree of novelty and a different perspective owing to a different population, we did not consider other factors that may impact DMHA utilization, such as user interface and engagement concerns [65]. These issues deserve further exploration, especially concerning how important engagement is to lower dropout rates of digital health mental apps [66].

Societal issues such as shame, fear, and stigma also merit further research. Even though we replicated Borghouts’ approach to stigma assessment for methodological replication purposes, that does not necessarily mean this theoretical construct (Depression Stigma Scale by Griffiths et al. [67]) was the most suitable one. Given that stigma showed no statistically significant association with DMHA use in all designed models, replicating these analyses with other validated stigma assessment tools (e.g., the Stigma Scale [68] or the Portuguese Version of the Stigma Scale [69]) on a nationwide survey might bring about further insights on the impact of societal issues on DMHA use.

Lastly, the response rate was 1.7%. Despite this not being a typical response rate for web-based surveys among student populations [46], that is due to the number of potential respondents being so high compared with similar studies (ca. 4,000 students). Nonetheless, the potential for response bias and self-selection remains, and there may be differences between non-responders and responders.

## Conclusions

This study focused on the academic community of the University of Porto and found four factors associated with DMHA use. Our results revealed that participants’ use of DMHA was directly associated with perceived stress and privacy concerns and a latent variable comprising both perceptions of mental health problems and coping strategies in place. All these issues and their implications must be factored into the decision to promote a DMHA-based approach to providing mental health resources to the academic community, and have implications for researchers and academia, industry, and policymakers concerning the adoption and implementation of digital mental health apps and associated interventions.

Regarding access barriers and their importance, developers should consider delivering these apps with low to no financial charges, offering evidence of their helpfulness, and focusing on timely care delivery. To increase DMHA use, there is a need to shape mindsets through mental health literacy, namely concerning discernment in terms of the seriousness of one’s conditions, acceptable stress levels, and overall behavior toward mental health. The results can inform the appropriate development and implementation of DMHA that meet the needs of the academic community.

### Supplementary Information

**Supplementary Material 1.**

**Supplementary Material 2.**

**Supplementary Material 3.**

**Supplementary Material 4.**

**Supplementary Material 5.**

**Supplementary Material 6.**

## Data Availability

The datasets used and/or analysed during the current study are available from the corresponding author on reasonable request.

## Abbreviations

DALY: Disability-Adjusted Life Years
DHMA: Digital Mental Health Applications/Apps
EU: European Union
OECD: Organisation for Economic Co-operation and Development
