# Supplementary material for: Individuals’ attitudes toward digital mental health apps and implications for adoption in Portugal: web-based survey
Source: BMC Med Inform Decis Mak. 2024 Apr 18;24:99. doi: 10.1186/s12911-024-02488-1 (PMC11025147; doi:10.1186/s12911-024-02488-1)
Supplement: Supplementary file 2 — Supplementary Material 2. [file 12911_2024_2488_MOESM2_ESM.pdf]

Survey - Academic Community

Survey on How Mobile Technologies Can Fulfill the Unmet Mental Health and Well-being Needs of the Academic Community of the University of Porto

The purpose of this survey is to understand the unmet mental health needs of the academic community (i.e., students, faculty, and staff) and the strategies they currently use to address their mental health, with in order to provide adequate resources (including digital tools). Previous research has revealed that university students encounter mental health problems and lack of wellbeing that are often not identified and treated, and that better access to targeted mental health resources is needed. However, there is a lack of research on the mental health needs of university students and the availability and use of technology to meet those needs. Furthermore, there is no study that addresses, in addition to the unmet needs of students, those of teachers and staff. Although there is a consensus in the literature that adolescents and young adults are at greater risk of psychological and emotional disorders, a complete picture of the academic community cannot be constructed without these other two groups. Such an inclusion also has the advantage of generating an understanding that allows for an approximation to the reality of the Portuguese population, paying special attention to potential biases.

We intend to investigate the following questions:

(1) What are the current unmet mental health and well-being needs of the academic community?
(2) What are the current tools and strategies used to address health and well-being?
(3) How can digital tools, such as mobile apps, fulfill unmet needs?
(4) What factors might influence the adoption/development of mental health apps?

This survey is being conducted as part of a PhD thesis for the PhD Programme in Health Data Science at the FMUP. The person responsible for collecting data is the student Diogo Nogueira Leite (202002508), who can be contacted at up202002508@up.pt for further questions.

This survey is comprised of 60 question(s).

Demographic and basic information

This part of the survey is about your basic and demographic information. This data is exclusively used for the characterisation of the sample of answers and will not be used individually or for purposes other than global characterisation.

[Q1] How old are you? (Please only enter the number of years old you are. If you do not wish to answer, please enter 0) *

Please write your answer here:

[Q2]

What gender do you identify as? *

Please select only one of the following options:

- Male
- Female
- Transgender man
- Transgender woman
- Genderqueer/ Non-conforming / Non-binary
- Uncertain about gender identity
- I prefer not to answer.
- Other

[Q3]

Select the option that best describes your situation. (Select all that apply.) *

Please select all that apply:

- ☐ Full-time student in the first cycle of studies (Bachelor’s Degree)
- ☐ Full-time student in the second cycle of studies (Master’s Degree)
- ☐ Full-time student in post-graduate studies/specialisation
- ☐ Full-time student in the third cycle of studies (Doctoral Degree)
- ☐ Part-time student in the first cycle of studies
- ☐ Part-time student in the second cycle of studies
- ☐ Part-time student in post-graduate studies/specialisation
- ☐ Part-time student in the third cycle of studies
- ☐ Professor (regardless of the professional category)
- ☐ Staff of the University of Porto and/or its Organic Units
- ☐ I prefer not to answer
- ☐ Other: ____________

[Q4] Please select the option that best describes your occupational status. *

Please select only one of the following options:

- ○ Full-time employee
- ○ Part-time employee
- ○ Full-time student (includes scholarship holders)
- ○ Student worker
- ○ Unemployed, looking for a job
- ○ Unemployed, not looking for a job
- ○ I prefer not to answer
- ○ Other ____________

[Q5]

When do you attend most of your classes at the University of Porto? (If you are not a student or you are, e.g., working on a thesis without a curricular component, please select "Not applicable") *

Please select only one of the following options:

- ◯ Daytime
- ◯ Evening
- ◯ Not applicable
- ◯ I prefer not to answer
- ◯ Other

[Q6]

What is your ethnicity? (Please select one answer. If you have multiple ethnicities, select the 'more than one ethnicity' option. Categorisation based on Census 2021) * *

Please select only one of the following options:

- ◯ White/White Portuguese/of European origin
- ◯ Black/Black Portuguese/Afro-descendant/of African origin.
- ◯ Asian/Portuguese of Asian origin/of Asian origin
- ◯ Gypsy/Portuguese Gypsy/Roma
- ◯ More than one ethnicity
- ◯ I prefer not to answer
- ◯ Other

[Q7]

Please select the option that best describes your current marital status. *

Please select only one of the following options:

- ◯ Single
- ◯ In a committed relationship, but not married
- ◯ Married
- ◯ Widower
- ◯ Divorced
- ◯ Separated
- ◯ I prefer not to answer
- ◯ Other ____________

[Q8]

Do you have children or dependents? *

Please select only one of the following options:

- ◯ Yes
- ◯ No
- ◯ I prefer not to answer

[Q9]

What is your current housing situation? (Select one answer - if in doubt, consider the situation you have lived in the longest) * *

Please select only one of the following options:

- ◯ I live alone
- ◯ I live with my partner or spouse
- ◯ I live with roommates
- ◯ I live with my family
- ◯ I prefer not to answer
- ◯ Other ________

[Q10]

Are you currently homeless? *

Please select only one of the following options:

- ◯ Yes
- ◯ No
- ◯ I prefer not to answer

[Q11]

What is the current annual income of your household?

*

Please select only one of the following options:

- ◯ Less than €5,000 (ca. €416/month)
- ◯ €5,001-10,000 (€417-833/month)
- ◯ € 10,001 – 13,500 (€834-1,125/month)
- ◯ € 13,501 – 19,000 (€1,126-1,583/month)
- ◯ € 19,001 – 27,500 (€1,584-2,291/month)
- ◯ € 27,501 – 32,500 (€2,292-2,708/month)
- ◯ € 32,501 – 40,000 (€2,709-3,333/month)
- ◯ € 40,001 – 50,000 (€3,334-4,166/month)
- ◯ € 50,001 – 100,000 (€4,167-8,333/month)
- ◯ € 100,001 – 250,000 (€8,334-20,833/month)
- ◯ More than €250,000 (>€20,833/month)
- ◯ I don’t know
- ◯ I prefer not to answer

[Q12]

Do you have a disability?

For the purposes of this survey, disability is defined as a mental or physical health condition that has lasted for more than 6 months and which limits major life activities, but which is not the result of a serious mental illness… *

Please select only one of the following options:

- ◯ Yes
- ◯ No
- ◯ I prefer not to answer

[Q12a]

What type of disability do you have? (Please select all that apply.)

For the purposes of this survey, disability is defined as a mental or physical health condition that has lasted for more than 6 months and which limits major life activities, but which is not the result of a serious mental illness… *

Answer this question only if the following conditions are true:
The answer is 'Yes' in question '12 [Q12]' (Do you have a disability? For the purposes of this survey, disability is defined as a mental or physical health condition that has lasted for more than 6 months and which limits major life activities, but which is not the result of a serious mental illness.)

Please select only one of the following options:

- ◯ Mild to moderate mental disability
- ◯ Physical/Mobility disability
- ◯ Chronic health problem (including chronic pain, cancer pathology, neurological pathology)
- ◯ Visual impairment
- ◯ Hearing impairment
- ◯ I prefer not to answer
- ◯ Other [ ]

Use of technology in general and of smartphone

This section is about the use of technology and smartphone.

[Q13] Which of the following devices do you use? (Select all that apply.) *

 Select all that apply

Please select all that apply:

- ☐ Computer (laptop or desktop)
- ☐ Smartphone
- ☐ Tablet
- ☐ Mobile phone, but not a smartphone
- ☐ Wearables (smartwatches, activity bracelets, etc.)
- ☐ I don’t use any of the previous options
- ☐ I prefer not to answer

[Q13a]

On a regular day, how often do you use a computer or mobile device to access social media?

*Social media can include Facebook, Instagram, Twitter, Snapchat, LinkedIn, TikTok, Pinterest, Telegram, WeChat, Weibo, etc. **

Answer this question only if the following conditions are true:

-------- Scenario 1 --------

The answer is in question '14 [Q13]' (Which of the following device(s) do you use? (Select all that apply.))

-------- or Scenario 2 --------

The answer is in question '14 [Q13]' (Which of the following device(s) do you use? (Select all that apply.))

-------- or Scenario 3 --------

The answer is in question '14 [Q13]' (Which of the following device(s) do you use? (Select all that apply.))

 Select one of the following answers
Please select only one of the following options:

- ◯ Almost constantly (on average more than 6 hours a day)
- ◯ Many times a day (on average between 4 and 6 hours a day)
- ◯ Occasionally (on average between 2 and 4 hours a day)
- ◯ A few times a day (on average between 0 and 2 hours a day)
- ◯ I don’t use social media
- ◯ I prefer not to answer

[Q13b] If you have a smartphone, what activities do you use it for? *

Answer this question only if the following conditions are true:
The answer is in question '14 [Q13]' (Which of the following device(s) do you use? (Select all that apply.))

 Select all that apply
Please select all that apply:

- ☐ Communication (calls, text messages...)
- ☐ Entertainment
- ☐ Games
- ☐ Social media
- ☐ E-mail
- ☐ School / homework
- ☐ News / current events
- ☐ Travel information / navigation
- ☐ Apps to track nutrition / diet
- ☐ Other health apps (e.g., chronic illness)
- ☐ Fitness / exercise
- ☐ Mental health
- ☐ I prefer not to answer
- ☐ Other: ____________

[Q13c] If you have a smartphone, is having enough space to download apps a concern? *

Answer this question only if the following conditions are true:
The answer is in question '14 [Q13]' (Which of the following device(s) do you use? (Select all that apply.))

 Select one of the following answers
Please select only one of the following options:

- ◯ Yes.
- ◯ No
- ◯ I don’t know
- ◯ I prefer not to answer.

[Q14] People can use the internet to watch videos/listen to music, play games, go on social media, use apps, research something, etc. on a computer or mobile phone or device.

On a regular day, how regularly do you proactively use the internet (excluding, for example, having messaging apps turned on but not being actively used)? *

 Select one of the following answers
Please select only one of the following options:

- ◯ Almost constantly (on average more than 6 hours a day)
- ◯ Many times a day (on average between 4 and 6 hours a day)
- ◯ Occasionally (on average between 2 and 4 hours a day)
- ◯ A few times a day (on average between 2 and 4 hours a day)
- ◯ I don’t use the Internet on a regular day
- ◯ I prefer not to answer

[Q15] Where do you most often access the Internet? *

 Select one of the following answers
Please select only one of the following options:

- At home
- At work
- At university
- On your mobile phone (wherever you are)
- In daily commuting (public transport, traffic, etc.)
- I prefer not to answer
- Other

[Q16] Do you have consistent access to Wi-Fi or mobile networks? *

 Select one of the following answers
Please select only one of the following options:

- Yes
- No
- I don’t know
- I prefer not to answer

[Q17] Do you have a mobile data plan? *

 Select one of the following answers
Please select only one of the following options:

- Yes
- No
- I don’t know
- I prefer not to answer

[Q17a] Do you worry about your mobile data plan when you use your phone (e.g., worry that you will run out of data, that an app will use up too much data, etc.)? *

Answer this question only if the following conditions are true:
The answer is 'Yes' to question '21 [Q17]' (Do you have a mobile data plan?)

 Select one of the following answers
Please select only one of the following options:

- Yes
- No
- I don’t know
- I prefer not to answer

[Q18] When you don't have access to Wi-Fi, do you switch on data (3G, 4G, 5G)? *

 Select one of the following answers
Please select only one of the following options:

- Yes
- No
- I don’t know
- I prefer not to answer

Mental health mobile apps

This section is about technology specifically designed for mental health.

[Q19]

Have you ever used a mental health app? (Select one answer.)

When we say, 'mental health app', we mean an app on your mobile phone or tablet that helps you to manage your mental, emotional, or psychological health, or to access resources to support your mental, emotional, or psychological health. *

 Select one of the following answers
Please select only one of the following options:

- ◯ Yes, I am currently using a mental health app
- ◯ Yes, I have used a mental health app, but I don't use it anymore
- ◯ No, I have never used a mental health app but am interested in using one
- ◯ No, I have never used a mental health app and I am not interested in using one
- ◯ I prefer not to answer

[Q19a] The next set of statements will look at your views on the use of mental health apps. Please indicate the extent to which you agree (or disagree) with each of the statements, on a scale of 1 (Strongly disagree) to 5 (Strongly agree). *

Answer this question only if the following conditions are true:

-------- Scenario 1 --------

The answer is 'Yes, I currently use a mental health app' in question '24 [Q19]' (Have you ever used a mental health app? (Select one answer.) When we say, 'mental health app', we mean an app on your mobile phone or tablet that helps you to manage your mental, emotional, or psychological health, or to access resources to support your mental, emotional, or psychological health).

-------- or Scenario 2 --------

The answer is 'Yes, I have used a mental health app, but I don’t use it anymore’ in question '24 [Q19]' (Have you ever used a mental health app? (Select one answer.) When we say, 'mental health app', we mean an app on your mobile phone or tablet that helps you to manage your mental, emotional, or psychological health, or to access resources to support your mental, emotional, or psychological health).

Please select the appropriate position for each element:

	Strongly disagree (1)	Disagree (2)	I don’t know (3)	Agree (4)	Strongly agree (5)	I prefer not to answer
I consider that mental health mobile apps can be useful in my daily life.	○	○	○	○	○	○
I think using mental health mobile apps increases my chances of achieving goals that are important to me.	○	○	○	○	○	○

	Strongly disagree (1)	Disagree (2)	I don’t know (3)	Agree (4)	Strongly agree (5)	I prefer not to answer
I think using mental health apps helps me achieve my goals faster.	◯	◯	◯	◯	◯	◯
I think using mental health apps increases my productivity.	◯	◯	◯	◯	◯	◯

[Q19b] The next set of statements will look at your views on how your personal data may be used by a mental health app. Please indicate the extent to which you agree (or disagree) with each of the statements, on a scale from 1 (Strongly disagree) to 5 (Strongly agree). *

Only answer this question if the following conditions are true:

The answer is 'Yes, I currently use a mental health app' or 'Yes, I have used a mental health app, but I don't use it anymore' in question '24 [Q19]' (Have you ever used a mental health mobile app? (Select one answer.) When we say, 'mental health app', we mean an app on your mobile phone or tablet that helps you to manage your mental, emotional, or psychological health, or to access resources to support your mental, emotional, or psychological health).

Please select the appropriate position for each element:

	Strongly disagree (1)	Disagree (2)	I don't know (3)	Agree (4)	Strongly agree (5)	I prefer not to answer
I feel that using mental health mobile apps exposes me to others, making me uncomfortable.	○	○	○	○	○	○
I believe that the use of mental health mobile apps increases the availability of information about myself that I consider private and becomes available to others.	○	○	○	○	○	○
I feel that by using mental health mobile apps, information about me is disclosed which, if used, could compromise my privacy.	○	○	○	○	○	○

	Strongly disagree (1)	Disagree (2)	I don’t know (3)	Agree (4)	Strongly agree (5)	I prefer not to answer
I am concerned that my personal data placed on mental health apps may be used for other purposes without notifying me or asking for my permission.	◯	◯	◯	◯	◯	◯
When I give personal information to use mental health apps, I am concerned that it may use my information for other purposes.	◯	◯	◯	◯	◯	◯
I am concerned that mental health apps may share personal information with other groups without my permission for other purposes.	◯	◯	◯	◯	◯	◯

[Q19c]

The next set of statements will look at your views on how your personal data may be used by a mental health app. Please indicate the extent to which you agree (or disagree) with each of the statements, on a scale from 1 (Strongly disagree) to 5 (Strongly agree). *

Only answer this question if the following conditions are true:

The answer is 'No, I have never used a mental health app, but I am interested in using it' or 'No, I have never used a mental health app and I am not interested in using it' in question '24 [Q19]' (Have you ever used a mental health mobile app? (Select one answer.) When we say, 'mental health app', we mean an app on your mobile phone or tablet that helps you to manage your mental, emotional, or psychological health, or to access resources to support your mental, emotional, or psychological health).

Please select the appropriate position for each element:

	Strongly disagree (1)	Disagree (2)	I don’t know (3)	Agree (4)	Strongly agree (5)	I prefer not to answer
I feel that if I used mental health mobile apps, others would know more about me than what I am comfortable with.	○	○	○	○	○	○
I believe that if I used mental health mobile apps, information about myself that I consider private would be more easily available to others than I would like.	○	○	○	○	○	○

	Strongly disagree (1)	Disagree (2)	I don’t know (3)	Agree (4)	Strongly agree (5)	I prefer not to answer
I feel that if I used mental health mobile apps, information about me would be disclosed which, if used, would compromise my privacy.	◯	◯	◯	◯	◯	◯
I am concerned that mental health mobile apps may use my personal information for other purposes without notifying me or asking for my permission.	◯	◯	◯	◯	◯	◯
If I were to give personal information to use mental health apps, I am concerned that it may use my information for other purposes.	◯	◯	◯	◯	◯	◯
I am concerned if I were to use mental health apps that they may share my personal information with other groups without getting my authorization.	◯	◯	◯	◯	◯	◯

[Q20]

The next set of statements will look at your views on mental health apps. Please indicate the extent to which you agree (or disagree) with each of the statements, on a scale of 1 (Strongly disagree) to 5 (Strongly agree). *

Please select the appropriate position for each element:

	Strongly disagree (1)	Disagree (2)	I don't know (3)	Agree (4)	Strongly agree (5)	I prefer not to answer
I feel that the people who are important to me think I should use mental health mobile apps.	○	○	○	○	○	○
People who influence my behavior think that I should use mental health apps.	○	○	○	○	○	○
People whose opinions I value prefer that I use mental health apps.	○	○	○	○	○	○
I consider that I have the necessary knowledge to use mental health mobile applications.	○	○	○	○	○	○
I consider that I have the resources (economic, technological, etc.) necessary to use mental health mobile applications.	○	○	○	○	○	○

I find that mental health mobile apps are compatible with other technologies I use.	○	○	○	○	○	○
I find it easier to get help when I need it using mental health mobile apps.	○	○	○	○	○	○

[Q21] When thinking about using mental health mobile apps, what aspects are important to you? *

□ Select all that apply

Please select **all** that apply:

- ☐ Available in my mother tongue
- ☐ Free of charges
- ☐ Adaptation of the application to my culture and its specificities
- ☐ The application allows interaction with other people
- ☐ The people I interact with in the app have the same cultural background as me
- ☐ The people I interact with on the app have similar mental health experiences to me
- ☐ Ensuring the privacy of my personal information
- ☐ The application does not have a negative effect on my device (e.g. it does not consume too much phone battery or take up too much memory)
- ☐ Existence of parts of the application that can be used offline
- ☐ Possibility that the application can be easily used by people with visual impairments
- ☐ Possibility that the application can be easily used by people with hearing disabilities
- ☐ I prefer not to answer
- ☐ Other: ____________

Healthcare use and resources

This section is about healthcare use and resources.

[Q22]

Do you currently have a health insurance (e.g., ADSE, Médis, Multicare, etc.)? *

 Select one of the following answers
Please select only one of the following options:

- Yes
- No
- I don’t know
- I prefer not to answer

[Q22a] Do you know if your health insurance plan would cover an appointment with a mental health professional (psychiatrist, psychologist, nurse, etc.)? *

Answer this question only if the following conditions are true:
If the answer is 'Yes' to question '30 [Q22]' (Do you currently have a health insurance (e.g., ADSE, Médis, Multicare, etc.)?

 Select one of the following answers
Please select only one of the following options:

- No, for sure
- I don't think so, but I'm not sure
- I don’t know
- I think so, but I’m not sure
- Yes, sure
- I prefer not to answer

[Q23] Has there ever been a time during the last 12 months when you felt you might need help from a professional because of problems with your mental health, anxiety-provoking emotions or situations, or alcohol or drug use / other addictions? *

Select one of the following answers
Please select only one of the following options:

- ◯ Yes
- ◯ No
- ◯ I don’t know
- ◯ I prefer not to answer

[Q23a]

What mental or psychological health conditions, if any, have you faced in the last 12 months? (Select all that apply.) If the options do not mirror your mental health problems, feel free to describe the problem in your own words, by selecting the option "Other". If you prefer not to answer, select the "I prefer not to answer" option. *

Only answer this question if the following conditions are true:

The answer is 'Yes' or 'I don't know' in question '32 [Q23]' (Has there ever been a time during the last 12 months when you felt you might need help from a professional because of problems with your mental health, anxiety-provoking emotions or situations, or alcohol or drug use / other addictions?)

 Select all that apply.
Please select all that apply:

- ☐ Depression
- ☐ Anxiety
- ☐ Stress
- ☐ Difficulty in sleeping
- ☐ Loneliness
- ☐ Substance and/or alcohol abuse and/or other addictive behaviours
- ☐ Eating disorder
- ☐ Obsessions and compulsions that interfere with daily activities
- ☐ Mental health problems after childbirth
- ☐ Life event (e.g., change, death, illness)
- ☐ Interpersonal relationships (e.g., ended a relationship, had an argument with my father/mother)
- ☐ Psychotic events
- ☐ Schizophrenia
- ☐ Bipolar disorder
- ☐ I have not faced any mental or emotional health problems in the last 12 months
- ☐ I prefer not to answer
- ☐ Other: ____________

[Q24]

In the last 12 months, have you had any appointments with your GP or general practitioner about problems related to your mental health, emotions or anxiety-provoking emotions or situations, or alcohol or drug use/other addictions? *

 Select one of the following answers

Please select only one of the following options:

- ◯ Yes
- ◯ No
- ◯ I don’t know
- ◯ I prefer not to answer

[Q25]

In the last 12 months, have you had any appointment with any other professional such as a psychiatrist or a psychologist, due to problems with your mental health, anxiety-provoking emotions or situations, or alcohol or drug use/other addictions? *

 Select one of the following answers

Please select only one of the following options:

- ◯ Yes
- ◯ No
- ◯ I don’t know
- ◯ I prefer not to answer

[Q25a]

Have you sought help for your mental or emotional health or a problem with alcohol, drugs, or other addictions? If yes, for which (Select all that apply.)? *

Answer this question only if the following conditions are true:

-------- Scenario 1 --------

The answer is 'Yes' to question '34 [Q24]' (In the last 12 months, have you had an appointment with your GP or general practitioner about problems related to your mental health, anxiety-provoking emotions or situations, or alcohol or drug use/other addictions?)

-------- or Scenario 2 --------

The answer is 'Yes' to question '35 [Q25]' (In the last 12 months, have you had any appointment with any other professional such as a psychiatrist or a psychologist, due to problems with your mental health, anxiety-provoking emotions or situations, or alcohol or drug use/other addictions?)

Select all the options that apply

Please select all that apply:

- ☐ Depression
- ☐ Anxiety
- ☐ Stress
- ☐ Difficulty in sleeping
- ☐ Loneliness
- ☐ Substance and/or alcohol abuse and/or other addictive behaviours
- ☐ Eating disorder
- ☐ Obsessions and compulsions that interfere with daily activities
- ☐ Mental health problems after childbirth
- ☐ Life event (e.g., change, death, illness)
- ☐ Interpersonal relationships (e.g., ended a relationship, had an argument with my father/mother)
- ☐ Psychotic events
- ☐ Schizophrenia
- ☐ Bipolar disorder
- ☐ I have not faced any mental or emotional health problems in the last 12 months

- ☐ I don’t know.
- ☐ I prefer not to answer
- ☐ Other: ____________

[Q26]

In the last 12 months, have you tried to get help from any digital resource, including apps or messaging services, for problems with your mental health, emotions, or use of alcohol, drugs, or other addictions? *

 Select one of the following answers
Please select **only one** of the following options:

◯ Yes

No

◯ I prefer not to answer

[Q26a]

How useful did you feel this digital resource was? *

Only answer this question if the following conditions are true:

The answer is 'Yes' to question '38 [Q26]' (In the last 12 months, have you tried to get help from any digital resource, including apps or messaging services, for problems with your mental health, emotions, or use of alcohol, drugs, or other addictions?)

 Select one of the following answers
Please select only one of the following options:

◯ Very useful

◯ Quite useful

◯ Reasonably useful

◯ Somewhat useful

◯ Not useful

◯ I prefer not to answer

[Q26b]

What was the main reason you did not try to get help from a digital resource, including apps or messaging services? *

Only answer this question if the following conditions are true:

The answer is 'No' in question '38 [Q26]' (In the last 12 months, have you tried to get help from any digital resource, including apps or messaging services, for problems with your mental health, emotions, or use of alcohol, drugs, or other addictions?)

Select one of the following answers

Please select only one of the following options:

- ◯ I felt better / no longer needed
- ◯ I wanted to solve the problem by myself
- ◯ I don't own a smartphone or a computer
- ◯ I did not know these apps
- ◯ I don’t trust apps
- ◯ Privacy and data security issues
- ◯ I didn't think it would be useful / wouldn't work
- ◯ It was very expensive
- ◯ I don’t have time for it
- ◯ I was already receiving traditional / face-to-face clinical follow-up
- ◯ I thought it was not necessary
- ◯ I don't have enough space to download new apps
- ◯ I prefer not to answer
- ◯ Other ____________

[Q27] In the last 12 months, have you had online contact with people who have mental health problems or use alcohol, drugs, or other addictions similar to yours through means such as social media, blogs, or online forums? *

 Select one of the following answers
Please select only one of the following options:

- ◯ Yes
- ◯ No
- ◯ I prefer not to answer

[Q28]
In the last 12 months, have you used any digital resources to find, contact or be referred to a mental health professional?

For example, by SMS, online message, video chat or a mental health or health-related app.* *

 Select one of the following answers
Please select **only one** of the following options:

- ◯ Yes
- ◯ No
- ◯ I prefer not to answer

[Q29]
If you needed to seek out resources for your mental health as a member of the academic community at the University of Porto, would you know where to go*? *

 Select one of the following answers
Please select **only one** of the following options:

- ◯ Yes
- ◯ No
- ◯ I prefer not to answer

[Q30]

Have you used any of these resources from the University of Porto? *

 Select all that apply
Please select all that apply:

- [ ] Psychological counselling
- [ ] SASUP – *Serviços de Ação Social da Universidade do Porto*
- [ ] Wellness and Mental Health Workshops (e.g., wellness and mental health at work, anxiety management, work-life balance)
- [ ] Face-to-Face Workshops (e.g., time management, mindfulness, avoiding burnout, self-care)
- [ ] I did not use any resources from the University of Porto
- [ ] I prefer not to answer
- [ ] Other: ________

[Q31]

Who do you usually talk to when you feel sad, anxious, worried, or stressed? *

 Select all the options that apply
Please select **all** that apply:

- [ ] Friend(s)
- [ ] Partner
- [ ] Family member(s)
- [ ] Professor(s)
- [ ] Coworker(s)
- [ ] Health professional(s)
- [ ] SASUP – *Serviços de Ação Social da Universidade do Porto*
- [ ] I don't talk to anyone
- [ ] I prefer not to answer

[Q32]

Which of the following resources and strategies do you currently use to cope with your mental and/or psychological health (e.g., when you feel sad, anxious, worried, or stressed? *

 Select all that apply

Please select all that apply:

- [ ] Informal support, such as talking or spending time with family or friends
- [ ] Informal support through the use of pets (e.g., dogs, cats, etc.)

- [ ] Professional services (e.g., appointment with a psychologist or psychiatrist)
- [ ] Social media (e.g., Facebook, Instagram, Twitter, Reddit)
- [ ] Online forums or communities (e.g., Mental Health Forum, BeyondBlue, SMS eHealth)

- [ ] Websites (e.g., Psic.ON - Online Psychological Support from UPorto, saudemental.pt, eusinto.me)
- [ ] Apps (e.g., Headspace, 29k FJN, Hug-a-Group)
- [ ] SNS24 Psychological Counselling Line (808 24 24 24).
- [ ] Other telephone or text helplines (e.g., SOS Voz Amiga, Conversa Amiga)

- [ ] Physical exercise
- [ ] Writing
- [ ] Painting, drawing, colouring, photographing, etc.
- [ ] Doing handicrafts, sewing, etc.
- [ ] Listening to music
- [ ] Playing an instrument, singing, or composing
- [ ] Reading
- [ ] Cooking
- [ ] Playing games
- [ ] I currently do not use any resources or strategies to deal with my mental and/or psychological health
- [ ] I prefer not to answer
- [ ] Other: ______

[Q32a]

Please indicate, for each resource you currently use, how useful that resource is for maintaining your mental health, on a scale of 1 (Not at all useful) to 5 (Extremely useful). *

Answer this question only if the following conditions are true:

-------- Scenario 1 --------

The answer is in question '46 [Q32]' (Which of the following resources and strategies do you currently use to manage your mental and/or psychological health (e.g., when you feel sad, anxious, worried, or stressed)?

-------- or Scenario 2 --------

The answer is in question '46 [Q32]' (Which of the following resources and strategies do you currently use to manage your mental and/or psychological health (e.g., when you feel sad, anxious, worried, or stressed?))

Please select the appropriate position for each element:

	Not at all useful (1)	Somewhat useful (2)	Reasonably useful (3)	Very useful (4)	Extremely useful (5)	I prefer not to answer
Informal support, such as talking or spending time with family or friends	○	○	○	○	○	○
Professional services (examples: therapy with a psychologist or psychiatrist)	○	○	○	○	○	○
Social media (e.g., Facebook, Instagram, Twitter, Reddit)	○	○	○	○	○	○
Online forums or communities (e.g., Mental Health Forum, BeyondBlue, SMS eHealth)	○	○	○	○	○	○

	Not at all useful (1)	Somewhat useful (2)	Reasonably useful (3)	Very useful (4)	Extremely useful (5)	I prefer not to answer
Websites (e.g., Psic.ON - Online Psychological Support from UPorto, saudemental.pt, eusinto.me)	○	○	○	○	○	○
Apps (e.g., Headspace, 29k FJN, Hug-a-Group)	○	○	○	○	○	○
SNS24 Psychological Counselling Line (808 24 24 24)	○	○	○	○	○	○
Other telephone or text crisis helplines (e.g., SOS Friend Voice, Friend Talk)	○	○	○	○	○	○
Sports or physical activities	○	○	○	○	○	○
Writing	○	○	○	○	○	○
Painting, drawing, colouring, photographing, etc.	○	○	○	○	○	○
Doing handicrafts, sewing, etc.	○	○	○	○	○	○

	Not at all useful (1)	Somewhat useful (2)	Reasonably useful (3)	Very useful (4)	Extremely useful (5)	I prefer not to answer
Listening to music	○	○	○	○	○	○
Playing an instrument, singing, or composing	○	○	○	○	○	○
Reading	○	○	○	○	○	○
Cooking	○	○	○	○	○	○
Playing games	○	○	○	○	○	○
I do not currently use any resources or strategies to manage my mental health	○	○	○	○	○	○
I prefer not to answer	○	○	○	○	○	○

[Q33]

Which of the following resources and strategies would you like to use to address your mental health? *

□ Select all that apply

Please select all that apply:

- [ ] Informal support, such as talking or spending time with family or friends
- [ ] Informal support through the use of pets (e.g., dogs, cats, etc.)
- [ ] Professional services (e.g., appointments with a psychologist or psychiatrist)
- [ ] Social media (e.g., Facebook, Instagram, Twitter, Reddit)
- [ ] Online forums or communities (e.g., Mental Health Forum, BeyondBlue, SMS eHealth)
- [ ] Websites (e.g., Psic.ON - Online Psychological Support from UPorto, saudemental.pt, eusinto.me)
- [ ] Apps (e.g., Headspace, 29k FJN, Hug-a-Group)
- [ ] SNS24 Psychological Counselling Line (808 24 24 24)
- [ ] Other telephone or text helplines (e.g. SOS Voz Amiga, Conversa Amiga)
- [ ] Physical exercise
- [ ] Writing
- [ ] Painting, drawing, colouring, photographing, etc.
- [ ] Doing handicrafts, sewing, etc.
- [ ] Listening to music
- [ ] Playing an instrument, singing, or composing
- [ ] Reading
- [ ] Cooking
- [ ] Playing games
- [ ] I currently do not use any resources or strategies to deal with my mental and/or psychological health
- [ ] I prefer not to answer
- [ ] Other: ____________

[Q33a]

Please indicate, for each resource you would like to use but do not currently use, how likely you are to use that resource, on a scale of 1 (Extremely unlikely) to 5 (Extremely likely). *

Answer this question only if the following conditions are true:

-------- Scenario 1 --------

The answer is in question '48 [Q33]' (Which of the following resources and strategies would you like to use to manage your mental health?)

-------- or Scenario 2 --------

The answer is in question '48 [Q33]' (Which of the following resources and strategies would you like to use to manage your mental health?)

Please select the appropriate position for each element:

	Extremely unlikely (1)	Unlikely (2)	Neither likely nor unlikely (3)	Likely (4)	Extremely likely (5)	I prefer not to answer
Informal support, such as talking or spending time with family or friends	○	○	○	○	○	○
Professional services (e.g., therapy with a psychologist or psychiatrist)	○	○	○	○	○	○
Social media (e.g., Facebook, Instagram, Twitter, Reddit)	○	○	○	○	○	○
Online forums or communities (e.g., Mental Health Forum, BeyondBlue, SMS eHealth)	○	○	○	○	○	○

	Extremely unlikely (1)	Unlikely (2)	Neither likely nor unlikely (3)	Likely (4)	Extremely likely (5)	I prefer not to answer
Websites (e.g., Psic.ON - Online Psychological Support from UPorto, saudemental.pt, eusinto.me)	◯	◯	◯	◯	◯	◯
Apps (e.g., Headspace, 29k FJN, Hug-a-Group)	◯	◯	◯	◯	◯	◯
SNS24 Psychological Counselling Line (808 24 24 24)	◯	◯	◯	◯	◯	◯
Other telephone or text crisis lines (e.g., SOS Friend Voice, Friend Talk)	◯	◯	◯	◯	◯	◯
Exercise or physical activities	◯	◯	◯	◯	◯	◯
Writing	◯	◯	◯	◯	◯	◯
Painting, drawing, colouring, photographing, etc.	◯	◯	◯	◯	◯	◯
Doing handicrafts, sewing, etc.	◯	◯	◯	◯	◯	◯
Listening to music	◯	◯	◯	◯	◯	◯
Playing an instrument, singing, or composing	◯	◯	◯	◯	◯	◯

	Extremely unlikely (1)	Unlikely (2)	Neither likely nor unlikely (3)	Likely (4)	Extremely likely (5)	I prefer not to answer
Reading	◯	◯	◯	◯	◯	◯
Cooking	◯	◯	◯	◯	◯	◯
Playing games	◯	◯	◯	◯	◯	◯
I am not interested in using any resources or strategies to manage my mental health.	◯	◯	◯	◯	◯	◯
I prefer not to answer	◯	◯	◯	◯	◯	◯

[Q34]

When you think about using digital mental health resources, what things would you like to be able to do? *

☐ Select all that apply

Please select all that apply:

- ☐ Identify or recognise symptoms
- ☐ Monitoring symptoms
- ☐ Overcoming negative emotions and thoughts
- ☐ Talking to others for support
- ☐ Access to a mental health professional
- ☐ Read about other people's experiences of mental health
- ☐ Keep myself organized, and keep track of tasks and responsibilities
- ☐ Express myself or have a refuge related to art, photography or writing
- ☐ Distract myself from negative emotions and thoughts
- ☐ Obtaining information on symptoms and mental health conditions
- ☐ Obtain information on how to access local mental health resources
- ☐ Access educational materials on how to deal with stress
- ☐ Obtaining information on how to deal with grief or loss
- ☐ Obtaining information on how to deal with trauma
- ☐ Get information on how to deal with problems in the relationship.
- ☐ I prefer not to answer
- ☐ Other: ____________

[Q35]

What challenges, if any, do you face in accessing resources (e.g., information, clinical or support services, contact for human support, etc.) related to mental health?
(Select all that apply.) *

 Select all that apply
Please select all that apply:

☐ The waiting time to access resources is too long (access difficulties).

☐ The cost of accessing resources is too high (financial difficulties)
☐ I don’t have time
☐ I am worried/I worry about my privacy
☐ I am concerned that my actions are documented
☐ I am afraid of what others will think of me
☐ I do not feel that my problems are valued by my family, and I am afraid that someone will notify my parents/other relatives
☐ Service providers are not sufficiently sensitive to cultural and/or religious differences

☐ Service providers are not sufficiently sensitive to differences in sexual identity

☐ I have difficulties communicating in the official language of the country of residence
☐ I don't trust my ability to choose the right option
☐ I wonder if the services are useful
☐ I have had a bad experience with these services in the past
☐ The problem will improve on its own
☐ I believe my problems are not serious enough to merit these services

☐ I don't think anyone can understand my problems
☐ Stress is normal in the academic community
☐ I get a lot of support from other sources
☐ I faced no obstacles, difficulties or challenges
☐ I had no need to use the resources
☐ I prefer not to answer
☐ Other: ____________

[Q36] In what situations or moments do you most need support regarding your mental health? *

 Select all the options that apply
Please select all that apply:

- ☐ During the day
- ☐ At night
- ☐ At home
- ☐ At college
- ☐ At work
- ☐ Before or after a difficult exam
- ☐ Before or during a situation where I need to interact with others (events, presentations, etc.)
- ☐ When I have to deal with a significant life event (e.g., after a separation, death, or illness in my family)
- ☐ I prefer not to answer
- ☐ Other:

Experiences of stress, well-being, and mental health

This section is about stress, wellbeing, and mental health.

[Q37]

The next set of statements will look at how often you feel stress in a set of situations. Please indicate how often you feel distressed or anxious in each of the following situations, on a scale of 1 (Never) to 5 (Very often). *

Please select the appropriate position for each element:

	Never (1)	Rarely (2)	Occasionally (3)	Often (4)	Very often (5)	Not applicable	I prefer not to answer
Situations related to problems with personal relationships	◯	◯	◯	◯	◯	◯	◯
Situations related to family problems	◯	◯	◯	◯	◯	◯	◯
Situations related to work-related problems	◯	◯	◯	◯	◯	◯	◯
Situations related to financial problems	◯	◯	◯	◯	◯	◯	◯
Situations related to academic problems	◯	◯	◯	◯	◯	◯	◯
Situations related to domestic problems	◯	◯	◯	◯	◯	◯	◯
Situations when I am away from home	◯	◯	◯	◯	◯	◯	◯
Events that do not go as planned	◯	◯	◯	◯	◯	◯	◯

[Q38]

The following questions are about the month during the last 12 months when you felt the worst emotionally.

During that month, how often have your emotions interfered, on a scale of 1 (Never) to 5 (Very often), with... *

Please select the appropriate position for each element:

	Never (1)	Rarely (2)	Occasionally (3)	Often (4)	Very often (5)	Not applicable	I prefer not to answer
...your academic performance?	○	○	○	○	○	○	○
...your professional performance?	○	○	○	○	○	○	○
...your domestic chores?	○	○	○	○	○	○	○
…your social life?	○	○	○	○	○	○	○
...your relationship with friends and family?	○	○	○	○	○	○	○

[Q39]

The following questions will look at how you feel about different aspects of your life.

Please indicate for each of the following questions how often you feel this way, on a scale of **1 (Never) to 5 (Very often**).

How frequently... *

Please select the appropriate position for each element:

	Never (1)	Rarely (2)	Occasionally (3)	Often (4)	Very often (5)	Not applicable	I prefer not to answer
do you miss company?	◯	◯	◯	◯	◯	◯	◯
do you feel left out?	◯	◯	◯	◯	◯	◯	◯
do you feel isolated from others?	◯	◯	◯	◯	◯	◯	◯

[Q40]

The following questions will look at how you felt during the last 30 days.

Please indicate for each of the following questions how often you have felt this way in the last 30 days, on a scale of 1 (Never) to 5 (Always).

In the last 30 days, how often have you felt... *

Please select the appropriate position for each element:

	Never (1)	Rarely (2)	Occasionally (3)	Often (4)	Very often (5)	Not applicable	I prefer not to answer
...exhausted for no reason?	○	○	○	○	○	○	○
...nervous?	○	○	○	○	○	○	○
...so nervous that nothing could calm you down?	○	○	○	○	○	○	○
...hopeless?	○	○	○	○	○	○	○
...restless or agitated?	○	○	○	○	○	○	○
...so restless that you couldn't stand still?	○	○	○	○	○	○	○
...depressed?	○	○	○	○	○	○	○
...in constant effort (you felt that everything required effort)?	○	○	○	○	○	○	○
...so sad that nothing could cheer you up?	○	○	○	○	○	○	○
...worthless?	○	○	○	○	○	○	○

Perceptions of mental health

Many people have suffered (or are currently suffering) from a mental illness that affects their ability to be functional in a family, relational or professional context. Many different terms are used to refer to this condition: "mental illness", "mental health problem", "emotional disturbance", "psychological disturbance or disorder", "mental challenge", etc.

[Q41] Have you ever suffered from a mental health problem? *

 Select one of the following answers

Please select only one of the following options:

- ◯ Yes, I am currently suffering or have suffered
- ◯ No, never
- ◯ I prefer not to answer

[Q41a]

This question looks at the stigma associated with mental health. Stigma can make people feel bad about something that is out of their control. Please note that the following statements **do not** necessarily represent the project researchers' view of mental health or mental illness.

In this question, the term "mental health problem" will be used. However, other terms such as mental health, mental disorder, mental health condition, psychological health, emotional well-being, among others, may be used.

Please indicate the extent to which you agree (or disagree with) each of the following statements, on a scale of 1 (Strongly disagree) to 5 (Strongly agree): *****: *

Only answer this question if the following conditions are true:

The answer is 'Yes, I currently suffer or have suffered' in question '57 [Q41]' (Have you ever suffered from a mental health problem?).

Please select the appropriate position for each element:

	Strongly disagree (1)	Disagree (2)	I don't know (3)	Agree (4)	Strongly agree (5)	I prefer not to answer
I think the stereotypes about people with mental health problems apply to me	○	○	○	○	○	○
I think that, in general, I can live life the way I want to	○	○	○	○	○	○
I think negative stereotypes about people with mental health problems make me feel isolated	○	○	○	○	○	○

	Strongly disagree (1)	Disagree (2)	I don’t know (3)	Agree (4)	Strongly agree (5)	I prefer not to answer
I think I feel out of place/ left out from the world because I have a mental health problem	◯	◯	◯	◯	◯	◯
I think being surrounded by people who do not have a mental health problem makes me feel out of place or maladjusted	◯	◯	◯	◯	◯	◯
I don't think people without a mental health problem can understand me.	◯	◯	◯	◯	◯	◯
I think that because I have a mental health problem, no one would be interested in coming close to me	◯	◯	◯	◯	◯	◯
I think that because I have a mental health problem, I cannot contribute to society	◯	◯	◯	◯	◯	◯
I think I can lead a full and fulfilling life despite my mental health problem	◯	◯	◯	◯	◯	◯
I think that in my culture it is acceptable to receive treatment for mental health problems	◯	◯	◯	◯	◯	◯
I prefer not to answer	◯	◯	◯	◯	◯	◯

[Q42]

The next question will look at your views and opinions of people with a mental illness. In this section, the term 'mental health problem' will be used. However, other terms such as mental illness, mental disorder, psychological disorder, or mental health condition may be used.

Please indicate the extent to which you agree (or disagree with) each of the following statements, on a scale of 1 (Strongly disagree) to 5 (Strongly agree): *

Please select the appropriate position for each element:

	Strongly disagree (1)	Disagree (2)	I don't know (3)	Agree (4)	Strongly agree (5)	I prefer not to answer
Most people believe that people with a mental health problem could overcome the problem if they wanted to.	○	○	○	○	○	○
Most people believe that having a mental illness is a sign of personal weakness.	○	○	○	○	○	○
Most people believe that mental illness is not a real disease.	○	○	○	○	○	○
Most people believe that people with mental health problems are dangerous.	○	○	○	○	○	○
Most people believe that it is better to avoid people with a mental illness so that they don't become mentally ill too.	○	○	○	○	○	○

	Strongly disagree (1)	Disagree (2)	I don't know (3)	Agree (4)	Strongly agree (5)	I prefer not to answer
Most people believe that people with mental health problems are unpredictable.	◯	◯	◯	◯	◯	◯
Most people would not tell anyone if they had a mental health problem.	◯	◯	◯	◯	◯	◯
Most people would not hire someone if they knew they had a mental illness.	◯	◯	◯	◯	◯	◯
I think I can lead a full and fulfilling life despite my mental health problem.	◯	◯	◯	◯	◯	◯
Most people would not vote for a politician if they knew they had a mental illness.	◯	◯	◯	◯	◯	◯
I prefer not to answer.	◯	◯	◯	◯	◯	◯

[Q43]

What else would you like to tell us? This includes, but is not limited to, for example, sharing more information about your mental health or wellbeing, coping strategies and resources or use of (and interest in) technology.

If you prefer not to answer, you can leave this space blank.

Please write your answer here:

Should you wish to be contacted to receive a summary of the results arising from this survey in a published study, please complete this form (https://inqueritos.up.pt/index.php?r=survey/index&sid=489829&lang=pt).

Thank you for your participation and contribution to the research!

Thank you for completing this survey.
