# Supplementary material for: Individuals’ attitudes toward digital mental health apps and implications for adoption in Portugal: web-based survey
Source: BMC Med Inform Decis Mak. 2024 Apr 18;24:99. doi: 10.1186/s12911-024-02488-1 (PMC11025147; doi:10.1186/s12911-024-02488-1)
Supplement: Supplementary file 4 — Supplementary Material 4. [file 12911_2024_2488_MOESM4_ESM.docx]

| **Additional file 4. Comparative characterists of participants.** | | | | |
| --- | --- | --- | --- | --- |
| **Characteristics** | | **Value ^a^** | **Borghouts [43]** | **p-value** |
| **Age (years), mean (SD)** | | 24.3 (11) | 23.8 (8) | 0.391 ^b^ |
| **Gender^b^, n (%)** | |  |  |  |
|  | Male | 145 (26.9) | 171 (34.2) | 0.013 ^c^ |
|  | Female | 368 (68.3) | 314 (62.8) |  |
|  | Other | 26 (4.8) | - |  |
| **Enrollment status, n (%)** | |  |  |  |
|  | Full-time | 450 (83.5) | 306 (61.2) | <0.001 ^c^ |
|  | Part-time | 44 (8.2) | 167 (33.4) |  |
|  | Faculty | 38 (7.1) | - |  |
|  | Other | 15 (2.8) | - |  |
| **Employment status, n (%)** | |  |  |  |
|  | Unemployed | 391 (72.5) | 213 (42.6) | <0.001 ^c^ |
|  | Part-time | 67 (12.4) | 158 (31.6) |  |
|  | Full-time | 67 (12.4) | 63 (12.6) |  |
|  | Other | 14 (2.6) | 5 (1) |  |
| **Race, n (%)** | |  |  |  |
|  | White | 506 (93.9) | 137 (27.4) | <0.001 ^c^ |
|  | Asian | 3 (0.6) | 66 (13.2) |  |
|  | More than one race | 12 (2.2) | 44 (8.8) |  |
|  | Black | 1 (0.2) | 32 (6.4) |  |
|  | Other | 13 (2.4) | 3 (0.6) |  |
| **Marital status, n (%)** | |  |  |  |
|  | Single | 341 (63.3) | 289 (57.8) | 0.003 ^c^ |
|  | In a committed relationship | 120 (22.2) | 146 (29.2) |  |
|  | Married | 66 (12.2) | 35 (7) |  |
|  | Divorced or separated | 7 (1.3) | 9 (1.8) |  |
| **Children, n (%)** | |  |  |  |
|  | Yes | 71 (13.2) | 69 (13.8) | 0.838 ^c^ |
|  | No | 468 (86.8) | 416 (83.2) |  |
| **Living situation, n (%)** | |  |  |  |
|  | Live with family | 323 (59.9) | 389 (77.8) | <0.001 ^c^ |
|  | Live with spouse or partner | 70 (13.0) | 45 (9) |  |
|  | Live alone | 48 (8.9) | 18 (3.6) |  |
|  | Live with roommate(s) | 85 (15.8) | 14 (2.8) |  |
|  | Other | 13 (2.4) | 10 (2) |  |
| **Homeless, n (%)** | |  |  |  |
|  | Yes | 2 (0.4) | 12 (2.4) | 0.010 ^c^ |
|  | No | 537 (99.6) | 472 (94.4) |  |
| **Household income (EUR €), n (%)** | |  |  |  |
|  | <10,000 | 77 (14.3) | - | - |
|  | 10,001-27,500 | 170 (31.5) | - |  |
|  | 27,501- 50,000 | 89 (16.5) | - |  |
|  | 50,001-100,000 | 27 (5.0) | - |  |
|  | 100,001 or above | 2 (0.3) | - |  |
| **Disability** | |  |  |  |
|  | Yes | 39 (7.2) | 47 (9.4) | 0.249 ^c^ |
|  | No | 486 (90.2) | 426 (85.2) |  |
| **Health insurance** | |  |  |  |
|  | Yes | 351 (65.1) | 402 (80.4) | <0.001 ^c^ |
|  | No | 188 (34.9) | 61 (12.2) |  |

a. Not all respondents answered each question; hence, some percentages do not sum to 100%.

b. Independent sample t-test.

c. Chi-square test.
