# Supplementary material for: Individuals’ attitudes toward digital mental health apps and implications for adoption in Portugal: web-based survey
Source: BMC Med Inform Decis Mak. 2024 Apr 18;24:99. doi: 10.1186/s12911-024-02488-1 (PMC11025147; doi:10.1186/s12911-024-02488-1)
Supplement: Supplementary file 5 — Supplementary Material 5. [file 12911_2024_2488_MOESM5_ESM.docx]

| **Additional file 5. Characteristics of users (current or past) and never users.** | | | | |
| --- | --- | --- | --- | --- |
| **Characteristics** | | **Users ^a^** | **Never users ^a^** | **p-value** |
| **Age (years), mean (SD)** | | 25.6 (12) | 20.9 (6) | <0.001 ^b^ |
| **Gender^b^, n (%)** | |  |  |  |
|  | Male | 26 (15.4) | 119 (32.2) | <0.001 ^c^ |
|  | Female | 125 (74.0) | 243 (65.7) |  |
|  | Other | 18 (10.7) | 8 (2.2) |  |
| **Enrollment status, n (%)** | |  |  |  |
|  | Full-time | 154 (91.1) | 295 (79.7) | 0.003 ^c^ |
|  | Part-time | 12 (7.1) | 30 (8.1) |  |
|  | Faculty | 3 (1.2) | 30 (8.1) |  |
|  | Other | - | 15 (4.1) |  |
| **Employment status, n (%)** | |  |  |  |
|  | Unemployed | 132 (78.1) | 259 (70.0) | 0.076 ^c^ |
|  | Part-time | 12 (7.1) | 55 (14.9) |  |
|  | Full-time | 20 (11.8) | 47 (12.7) |  |
|  | Other | 5 (3.0) | 9 (2.4) |  |
| **Race, n (%)** | |  |  |  |
|  | White | 158 (94.0) | 348 (94.8) | 0.679 ^c^ |
|  | Asian | - | 3 (0.8) |  |
|  | More than one race | 6 (3.6) | 6 (1.6) |  |
|  | Black | - | 1 (0.3) |  |
|  | Other | 4 (2.4) | 9 (2.5) |  |
| **Marital status, n (%)** | |  |  |  |
|  | Single | 113 (68.1) | 228 (62.0) | 0.003 ^c^ |
|  | In a committed relationship | 46 (27.7) | 74 (20.1) |  |
|  | Married | 7 (4.2) | 59 (16.0) |  |
|  | Divorced or separated | - | 7 (0.3) |  |
| **Children, n (%)** | |  |  |  |
|  | Yes | 163 (96.4) | 305 (82.4) | <0.001 ^c^ |
|  | No | 6 (3.6) | 65 (17.6) |  |
| **Living situation, n (%)** | |  |  |  |
|  | Live with family | 101 (59.8) | 222 (60.0) | 0.865 ^c^ |
|  | Live with spouse or partner | 20 (11.8) | 50 (13.5) |  |
|  | Live alone | 15 (8.9) | 33 (8.9) |  |
|  | Live with roommate(s) | 30 (17.8) | 55 (14.9) |  |
|  | Other | 3 (1.8) | 10 (2.7) |  |
| **Homeless, n (%)** | |  |  |  |
|  | Yes | - | 2 (0.5) | 0.945 ^c^ |
|  | No | 169 (100.0) | 362 (99.5) |  |
| **Household income (EUR €), n (%)** | |  |  |  |
|  | <10,000 | 34 (32.4) | 43 (16.5) | 0.023 ^c^ |
|  | 10,001-27,500 | 42 (40.0) | 128 (49.2) |  |
|  | 27,501- 50,000 | 21 (20.0) | 68 (26.2) |  |
|  | 50,001-100,000 | 8 (7.6) | 19 (7.3) |  |
|  | 100,001 or above | - | 2 (0.8) |  |
| **Disability** | |  |  |  |
|  | Yes | 18 (11.4) | 21 (5.7) | 0.022 ^c^ |
|  | No | 140 (88.6) | 348 (94.3) |  |
| **Health insurance** | |  |  |  |
|  | Yes | 101 (59.8) | 250 (67.6) | 0.078 ^c^ |
|  | No | 68 (40.2) | 120 (32.4) |  |

a. Past or current users and never users of digital mental health apps.

b. Independent sample t-test.

c. Chi-square test.
