# Supplementary material for: Individuals’ attitudes toward digital mental health apps and implications for adoption in Portugal: web-based survey
Source: BMC Med Inform Decis Mak. 2024 Apr 18;24:99. doi: 10.1186/s12911-024-02488-1 (PMC11025147; doi:10.1186/s12911-024-02488-1)
Supplement: Supplementary file 6 — Supplementary Material 6. [file 12911_2024_2488_MOESM6_ESM.docx]

| **Additional file 6. Overview of responses to survey items (N=539).** | | | | |
| --- | --- | --- | --- | --- |
| **Variable** | | **Value** | **Borghouts [43]** | **p-value** |
| **Technology ownership, n (%)** | | | | |
|  | Smartphone | 521 (96.7) | 443 (88.6) | <0.001 |
|  | Desktop or laptop computer | 524 (97.2) | 463 (92.6) | 0.001 |
|  | Tablet | 155 (28.8) | 138 (27.6) | 0.730 |
|  | Mobile/cell phone but not a smartphone | 20 (3.7) | 32 (6.4) | 0.065 |
| **Technology use, n (%)** | |  |  |  |
|  | Access to Wi-Fi | 509 (94.4) | 448 (89.6) | 0.006 |
|  | Access to a mobile data plan | 516 (95.7) | 440 (88) | <0.001 |
|  | Use of internet constantly or many times per day | 284 (52.7) | 452 (90.4) | <0.001 |
| **Mental health app use, n (%)** | |  |  |  |
|  | Current user | 32 (5.9) | 34 (6.8) | <0.001 |
|  | Past user | 137 (25.4) | 72 (14.4) |  |
|  | Nonuser, interested in using apps | 197 (36.6) | 199 (39.8) |  |
|  | Nonuser, not interested in using apps | 173 (32.1) | 180 (36) |  |
| **Mental illness (self-reported), n (%)** | |  |  |  |
|  | Yes | 265 (49.2) | 189 (37.8) | <0.001 |
|  | No | 225 (41.7) | 260 (52) |  |
|  | Prefer not to answer | 49 (9.1) | 51 (10.2) |  |
| **Most common mental health concerns, n (%)** | |  |  |  |
|  | Anxiety | 347 (64.4) | 207 (41.4) | <0.001 |
|  | Stress | 309 (57.3) | 219 (43.8) | <0.001 |
|  | Difficulty sleeping | 223 (41.4) | - | - |
|  | Depression | 164 (30.4) | 172 (34.4) | 0.193 |
| **Use of professional services in the past 12 months, n (%)** | |  |  |  |
|  | Yes | 234 (43.4) | 115 (23) | <0.001 |
|  | No | 305 (56.6) | 374 (74.8) |  |
| **Perceived need to seek help, n (%)** | |  |  |  |
|  | Yes | 71 (13.2) | 221 (44.2) | <0.001 |
|  | No | 468 (86.8) | 221 (44.2) |  |
|  | Prefer not to answer | 0 (0.0) | 21 (4.2) |  |
| **Perceived barriers to mental health resources use, n (%)** | |  |  |  |
|  | Financial reasons (e.g., too expensive) | 201 (37.3) | - | - |
|  | I question whether the resources are helpful | 145 (26.9) | - | - |
|  | I question how serious my needs are | 142 (26.3) | - | - |
|  | The waiting time to access resources is too long | 134 (24.9) | - | - |
|  | Stress is normal at college | 122 (22.6) | - | - |
|  | The problem will get better by itself | 104 (19.3) | - | - |
|  | I don’t have time | 87 (16.1) | - | - |
|  | I am concerned about privacy | 86 (16.0) | - | - |
| **Most important aspects of using mental health apps, n (%)** | |  |  |  |
|  | The app is free | 449 (83.3) | 429 (85.8) | 0.305 |
|  | Personal information will be kept private | 437 (81.1) | 397 (79.4) | 0.548 |
|  | Parts of the app can be used offline | 288 (53.4) | 256 (51.2) | 0.511 |
|  | No negative effect on device (e.g., drain phone battery) | 272 (50.5) | 264 (52.8) | 0.490 |
|  | Availability in maternal language | 247 (45.8) | - | - |
|  | People on the app have similar mental health experiences to mine | 160 (29.7) | 228 (45.6) | <0.001 |
| **Most common activities participants would like to do using mental health apps** | |  |  |  |
|  | Work through negative emotions and thoughts | 378 (70.1) | 329 (65.8) | 0.153 |
|  | Identify or recognize symptoms | 342 (63.5) | 290 (58) | 0.094 |
|  | Track symptoms | 296 (54.9) | 239 (47.8) | 0.026 |
|  | Distract myself from negative thoughts or emotions | 263 (48.8) | 228 (45.6) | 0.333 |
|  | Stay organized and keep on track of tasks and responsibilities | 261 (48.4) | - | - |
|  | Talk with other people to get/give support | 139 (25.8) | 244 (48.8) | <0.001 |
| **Stress score, mean (SD)** | | 21.0 (5.2)^a^ | 21.9 (5.3)^a^ | 0.006 |
| **Stigma score, mean (SD)** | | 23.9 (5.4)^b^ | 22.4 (6.2)^b^ | <0.001 |
| **Privacy score, mean (SD)** | | 17.2 (6.0)^c^ | 17.8 (7.6)^c^ | 0.157 |
| **Social influence score, mean (SD)** | | 6.2 (3.1)^d^ | 6.6 (3.4)^d^ | 0.047 |

a. The score could range from 7 to 35.

b. The score could range from 0 to 36.

c. The score could range from 6 to 30.

d. The score could range from 3 to 15.
