# Supplementary material for: Individuals’ attitudes toward digital mental health apps and implications for adoption in Portugal: web-based survey
Source: BMC Med Inform Decis Mak. 2024 Apr 18;24:99. doi: 10.1186/s12911-024-02488-1 (PMC11025147; doi:10.1186/s12911-024-02488-1)
Supplement: Supplementary file 5 [file 12911_2024_2488_MOESM3_ESM.doc]

Summary of modifications between survey questionnaires

Borghouts et al. (2021)	Nogueira-Leite et al. (2023)	Observations
Q6	Q1	
Q7	Q2	
Q8	Q3	
Q9	Q5	
Q10	Q6	
Q11	Non-existent	Question removed by suggestion of survey reviewers
Q12	Non-existent	Question removed by suggestion of survey reviewers
Q13	Non-existent	Question removed by suggestion of survey reviewers
Q15	Q13	
Q16	Q13a	
Q17	Q14	
Q18	Q15	
Q19	Q16	
Q20	Q13b	
Q21	Q17	
Q22	Q13c	
Q23	Q17a	
Non-existent	Q18	Question added by suggestion of survey reviewers
Q25	Q19	
Q26	Q19a	
Q27	Q20	
Q28	Q19b	
Q29	Q19c	
Q30	Q21	
Q32	Q22	
Q33	Q22a	
Q34	Q23	
Q35	Q23a	
Q36	Q24	
Q37	Q25	
Q38	Q25a	
Q39	Q25	
Q40	Q26	
Q41	Q26a	Answer options added by suggestion of survey reviewers
Q42	Q26b	
Q43	Q27	
Q44	Q28	
Q45	Q29	Answer options added or altered by suggestion of survey reviewers
Q46	Q30	
Q47	Q31	
Q48	Q32	
Q91	Q33	
Q49	Q32a	
Q50	Q33a	
Q58	Q34	
Q59	Q35	
Q60	Q36	
Q62	Q37	
Q63	Q38	
Q64	Q39	
Q65	Q40	
Q66	Q41	
Q67	Q41a	
Q68	Q42	
Q69	Q43	
Q71	Q4	
Q72	Q7	
Q73	Q8	
Q74	Q9	Answer option "I live with my children" removed due to survey reviewer suggestion
Q75	Q10	
Q76	Q11	
Q77	Non-existent	Question removed by suggestion of survey reviewers
Q78	Q12	
Q79	Q12a	
Q81	Non-existent	Question removed by suggestion of survey reviewers
Q82	Non-existent	Question removed by suggestion of survey reviewers
Q83	Non-existent	Question removed by suggestion of survey reviewers

Legend:

	Question wording adapted to Portuguese context
	Question and answers' wording adapted to Portuguese context
	Answer option wording adapted to Portuguese context
	Other changes - please see 'Observations' column

Remarks:

Questions from Borghouts et al. questionnaire not listed (eg. Q1 to 5) were not questions, but introductions to questions
